# Omega-3 Fatty Acids and Cancer Cell Cytotoxicity: Implications for Multi-Targeted Cancer Therapy

**DOI:** 10.3390/jcm5020015

**Published:** 2016-01-26

**Authors:** Donatella D’Eliseo, Francesca Velotti

**Affiliations:** 1 Department of Molecular Medicine, Istituto Pasteur-Fondazione Cenci Bolognetti, Sapienza University of Rome, 00161 Rome, Italy; donatella.deliseo@uniroma1.it; 2 Department of Ecological and Biological Sciences (DEB), La Tuscia University, Largo dell’Università, 01100 Viterbo, Italy

**Keywords:** fatty acids (FAs), *n-*3 polyunsaturated fatty acids (PUFAs), docosahexaenoic acid (DHA), eicosapentaenoic acid (EPA), apoptosis, cytotoxicity, cancer therapy, combinational therapy, drug resistance, cancer stem cells

## Abstract

Cancer is a major disease worldwide. Despite progress in cancer therapy, conventional cytotoxic therapies lead to unsatisfactory long-term survival, mainly related to development of drug resistance by tumor cells and toxicity towards normal cells. *n-*3 polyunsaturated fatty acids (PUFAs), eicosapentaenoic acid (EPA) and docosahexaenoic acid (DHA), can exert anti-neoplastic activity by inducing apoptotic cell death in human cancer cells either alone or in combination with conventional therapies. Indeed, *n-*3 PUFAs potentially increase the sensitivity of tumor cells to conventional therapies, possibly improving their efficacy especially against cancers resistant to treatment. Moreover, in contrast to traditional therapies, *n-*3 PUFAs appear to cause selective cytotoxicity towards cancer cells with little or no toxicity on normal cells. This review focuses on studies investigating the cytotoxic activity of *n-*3 PUFAs against cancer cells via apoptosis, analyzing the molecular mechanisms underlying this effective and selective activity. Here, we highlight the multiple molecules potentially targeted by *n-*3 PUFAs to trigger cancer cell apoptosis. This analysis can allow a better comprehension of the potential cytotoxic therapeutic role of *n-*3 PUFAs against cancer, providing specific information and support to design future pre-clinical and clinical studies for a better use of *n-*3 PUFAs in cancer therapy, mainly combinational therapy.

## 1. Introduction

Cancer is a major burden of disease worldwide and, in certain countries, it ranks the second most common cause of death following cardiovascular diseases [1]. Furthermore, as elderly people are most susceptible to cancer and population aging continues, cancer is projected to become the leading cause of death worldwide in many countries. Despite progress made in recent years in cancer therapy, traditional cytotoxic therapies such as chemo- and radio-therapy have multiple limitations, leading to treatment failure, cancer relapse and unsatisfactory long-term clinical results [2]. These limitations are mainly related to two important issues: (1) conventional therapies lead to development of drug resistance by tumor cells and/or fail to destroy cancer stem cells (CSCs) or tumor-initiating cells (TICs), a population of self-renewing and drug resistant cancer cells [3,4]; (2) conventional therapies can cause normal cells to die in massive number, leading to local and systemic toxicity. Since cancer cell survival is driven by complex molecular interactions between growth and death signals [5], most oncologists think that targeting a single molecular component may not be sufficient to disrupt this process and combinational therapies, targeting multiple molecules, pathways, or networks are needed to eradicate the tumor and increase patients' survival [6].

Omega-3 (ω-3 or *n-*3) fatty acids (FAs) are an important family of polyunsaturated fatty acids (PUFAs) and key nutrients, involved in normal growth and development of various human tissues [7,8,9]. Longer chain *n-*3 polyunsaturated fatty acids (PUFAs) are mainly composed of eicosapentaenoic acid (EPA) and docosahexaenoic acid (DHA). EPA has 20 carbon atoms and 5 double bonds (20:5*n-*3). DHA has a chain with 22 carbon atoms and 6 double bounds (22:6*n-*3), which makes it the longest chain and most unsaturated FA commonly found in biological systems. In the human body, DHA is either derived from β-oxidation of EPA or acquired from the diet. Cold-water oily fish are the main dietary source of essential *n-*3 PUFAs in humans, providing thus relatively large amount of EPA and DHA [10]. Beyond their role in physiological functions, *n-*3 PUFAs can affect some chronic diseases such as cancer [8,9,11,12,13]. Indeed, *n-*3 PUFAs or purified EPA and DHA can exert anti-neoplastic activity, playing a potential role either in cancer prevention or in cancer therapy [11,12,13].

Several decades ago, on the basis of human epidemiological studies, dietary oily fish and fish oil (FO) consumption have been associated with the protection against the development of some types of cancer, mainly colorectal, mammary and prostatic cancers [14,15]. Thereafter, most of the studies performed either *in vitro* or *in vivo* have demonstrated the protection by *n-*3 PUFAs against cancer risk. However, some reports question the effectiveness of these compounds in neoplastic prevention, and others argue that an increased *n-*3 PUFAs intake could induce some types of cancer [15,16,17,18,19]. Thus, the potential preventive role of *n-*3 PUFAs has become a subject of intense interest and debate. The biological effects of *n-*3 PUFAs on normal cells to prevent their transformation are not the topic of our dissertation, since exhaustive reviews have been written and have critically analyzed the data in the literature [15,16,20,21].

During recent years, extensive studies have also considered the potential therapeutic activity of *n-*3 PUFAs against established solid and hematological tumors [13,22]. A number of biological effects that could contribute to this activity have been suggested, including induced alteration by *n-*3 PUFAs of cancer cell invasion and metastasization, as well as proliferation and apoptosis [21,22,23,24,25]. The induction of tumor cell apoptosis plays an important role in cancer therapy and represents a prominent target of many treatment strategies. Several studies have demonstrated that *n-*3 PUFAs, EPA and DHA have inhibitory effects on tumor growth by inducing cancer cell death via apoptosis, either alone [22,23,24,25] or in combination with conventional anticancer therapies [26,27,28,29,30,31]. Although all these studies have proposed molecular mechanisms that account for the pro-apoptotic activity of *n-*3 PUFAs in cancer cells, the mechanisms are still not completely understood, and a large number of molecular targets of *n-*3 PUFAs have been identified and multiple mechanisms appear to underlie the induction of apoptosis by these FAs. However, notably, the cytotoxic activity exerted by *n-*3 PUFAs is very peculiar for two main reasons. First, it has the potential to increase the sensitivity of tumor cells to conventional cytotoxic therapies, possibly improving the efficacy of these therapies against some types of tumors, especially those otherwise resistant to treatments [26,27,28,29,30,31,32,33,34,35]. Second, it appears to be selective, in that *n-*3 PUFAs cause cytotoxicity against cancer cells with little or no toxicity on normal cells [28,36,37,38,39,40,41,42,43,44,45]. This is a very important point, since in order for a therapeutic agent to be truly effective, it should be toxic to cancer cells without harming normal cells; conversely, conventional chemotherapeutics kill cancer cells but also strike the healthy cells, causing adverse effects and severe morbidity. All the above considerations greatly support investigations carried out to assess the role of *n-*3 PUFAs as adjuvant, to improve the efficacy and tolerability of traditional anticancer therapies.

This review focuses on studies investigating the cytotoxic activity via apoptosis of *n-*3 PUFAs against cancer cells and analyzes the cellular and molecular mechanisms underlying this activity. In particular, it will be highlighted the wide range of molecules potentially targeted by *n-*3 PUFAs to induce cancer cell apoptosis. Firstly, in Section 2, it will be examined the pro-apoptotic activity exerted by *n-*3 PUFAs in different cancer models *in vitro* and *in vivo,* as well as the apoptotic pathways triggered by these FAs. Concerning this point, it will be also considered the important potential capability of EPA and DHA of inducing cytotoxicity towards drug-resistant cancer cells such as CSCs or TICs. Next, in Section 3, it will be analyzed the molecular events upstream the triggering of apoptosis by *n-*3 PUFAs, highlighting the multiple potential molecular targets of these FAs. This review could allow a better comprehension of the potential cytotoxic therapeutic role of the principal long chain *n-*3 PUFAs EPA and DHA against cancer, providing specific information and support to design future pre-clinical and clinical studies, which lead to the development of a more proper and effective use of these FAs in human cancer therapy, mainly combinational therapy.

## 2. Induction of Cancer Cell Apoptosis by *n-*3 Polyunsaturated Fatty Acids (PUFAs) and Triggering of the Intrinsic and Extrinsic Apoptotic Pathways

Apoptosis is a programmed cell death process, occurring in physiological and pathological conditions [46]**.** Caspases are central to apoptosis mechanism, as they are both the initiators and executioners of this process. There are three pathways by which caspases can be activated. The two commonly described initiation pathways are the intrinsic (or mitochondrial) and the extrinsic (or death receptor) apoptotic pathways. Both pathways eventually lead to a common pathway or the execution phase of apoptosis mediated by the executioner caspase-3, -6 and -7. A third initiation pathway is the intrinsic endoplasmic reticulum (ER) pathway [46,47]. The intrinsic or mitochondrial pathway is activated by endogenous stress signals such as growth factor deprivation, DNA-damaging chemicals and reactive oxygen species (ROS), which increase mitochondrial membrane permeability by modifying the interplay between B cell lymphoma protein*-*2 (Bcl-2) family proteins, that interact with mitochondrial membrane voltage-dependent anion channels. Bcl-2 family proteins have either pro-apoptotic (e.g., Bak, Bax, or Bok) or anti-apoptotic (e.g., Bcl-2, Bcl-xL, or Mcl-1) roles; a Bcl-2 subfamily, the BH3-only protein family (e.g., Bad, Bid, Bim, Noxa or Puma) also modulate pro- and anti-apoptotic Bcl-2 protein interactions. Pro-apoptotic stimuli shift the balance towards apoptic proteins, promoting the mitochondrial outer membrane permeabilization (MOMP), the subsequent release of cytochrome C into the cytosol, followed by its complex formation with procaspase-9 and apoptotic protease-activating factor 1 (APAF1), leading to the activation of the initiator caspase-9; then, caspase-9 activates the executioner caspases. The extrinsic pathway of apoptosis is activated by signal originated by death receptors such as TNFα-receptors, CD95 (Fas) and TNF-related apoptosis-inducing ligand (TRAIL)-receptors, following their interaction with their corresponding ligands, TNFα, FasL and TRAIL. Receptor activation leads to recruitment, to receptor associated lipid rafts, of adaptor molecules to form death-inducing signaling complexes (DISCs), which contains TNF receptor-associated death domain (TRADD), Fas-associated death domain (FADD), procaspase-8/FLICE and receptor-interacting protein kinase 1 (RIPK1). This complex induces the activation of caspase-8 and -10, which activate the executioner caspases. In addition, caspase-8 can also truncate Bid (tBid), which can migrate to the mitochondria to associate with Bax, increasing membrane permeability and converging thus to the activation of the intrinsic apoptotic pathway. The intrinsic ER pathway of apoptosis is activated in response to diverse arrays of stress such as oxidative stress, calcium influx and ER stress. The ER has three main functions: (1) folding, glycosylation and sorting of proteins to their proper destination; (2) synthesizing cholesterol and other lipids; and (3) maintenance of Ca^2+^ homeostasis. Disruption of any of these processes causes ER stress and activates the unfolded protein response (UPR). However, following prolonged ER stress, imbalanced calcium storage will activate calpain, which can inactivate Bcl-Xl and also activate the executioner caspases, leading to apoptosis. Finally, the apoptotic cascade is regulated by regulatory proteins, such as FLICE-like inhibitory proteins (FLIPs), which inhibit the extrinsic apoptotic pathway by binding to FADD and causing dissociation of the FADD/caspase-8 complex. Additionally, families of inhibitor of apoptosis protein (IAP) (e.g., XIAP, cIAP, and survivin) bind to caspase-3 and -9, thereby inhibiting caspase activity. Moreover, XIAP associated factor 1 (XAF1) negatively regulates the antiapoptotic function of XIAP.

Evasion of apoptosis by tumor cells is a hallmark of cancer [5] and defects in cancer cell apoptosis have been described at any point along the apoptotic pathways, including impaired receptor signaling, disrupted balance of anti- and pro-apoptotic Bcl-2 family proteins, reduced expression of caspases and increased expression of regulatory proteins (e.g., IAPs).

### 2.1. In Vitro and in Vivo Induction of Cancer Cell Apoptosis by n-3 PUFAs

*n-*3 PUFAs, EPA and DHA can induce apoptosis in tumor cells *in vitro* and *in vivo*, in a dose- and time-dependent manner. They induce apoptosis *in vitro,* in tumor cell lines derived from a wide range of solid tumors including colorectal carcinoma [37,48,49,50], esophageal [51] and gastric cancers [52], hepatocellular carcinoma [53,54,55], pancreatic cancer [56,57,58], cholangiocarcinoma [59], breast [60,61], ovarian [62], prostate [63,64] and bladder [65] cancers, neuroblastoma [66] and glioma [67], lung cancer [68,69], squamous cell carcinoma (SCC) [42] and melanoma [70,71]. Apoptosis induced by *n-*3 PUFAs, EPA and DHA has been also described in cancer cell lines derived from hematological tumors such as myeloid and lymphoid leukemias and lymphomas [72,73,74,75,76,77,78], as well as multiple myeloma [44,79].

In addition, in these last years a great attention has been given to CSCs or TICs, a small population of cancer cells with self-renewal and drug resistance properties, involved in cancer initiation, maintenance, metastasis and recurrence [2,3,4,80]. Resistance of CSCs/TICs to standard anti-cancer therapies is responsible for ineffectiveness of these treatments, leading to tumor recurrence and metastasis [2,3,4]. Therefore, in order to establish efficient therapeutic strategies that can prevent tumor relapse and induce a long-lasting clinical response, it is important to develop drugs that can specifically target and eliminate CSCs/TICs. Remarkably, recent *in vitro* studies have indicated the capability of *n-*3 PUFAs to affect colorectal and breast CSCs [81,82,83,84,85]. Indeed, it was shown that both EPA and DHA (10–70 μM), separately, induced apoptosis in cancer stem-like cells derived from the SW620 colon cancer cell line, and the effect was markedly increased when they acted simultaneously. Moreover, both compounds enhanced the efficacy of chemotherapeutics agents such as 5-fluorouracil (5-FU) and mitomycin C against the same target cells [82]. Accordingly, it was observed that EPA alone and (with increased efficacy) in combination with 5-FU + oxaliplatin (OX) (FuOX) induced apoptosis in FuOX-resistant HT-29 and HCT116 colorectal carcinoma cells, highly enriched in CSCs [83]. In addition, de Carlo *et al.* [84] found that 25 μM EPA induced the differentiation of colon CSCs, by upregulating cytokeratin 20 and mucin 2 and downregulating CD133 expression; they hypothesized that the increased degree of colon CSC differentiation could be strictly related to the EPA-induced sensitization of CD133^+^ cells to 5-FU. More recently, in human triple negative breast cancers, it has been shown that DHA inhibited mammosphere formation of TICs [85]. The capability of *n-*3 PUFAs to eliminate CSCs/TICs and/or increase their sensitivity to conventional antineoplastic drugs have a very important therapeutic potential, further supporting the anticancer use of these FAs as adjuvants in cancer therapies.

Suppression of tumor cell growth by *n-*3 PUFAs has been confirmed *in vivo*, in pre-clinical studies using cancer animal models mainly rapresented by transgenic “fat-1” mice (bearing the *Caenorhabditis elegans* “*n-*3 desaturase” gene able to convert *n-*6 to *n-*3 PUFAs, resulting thus in elevated *n-*3 PUFAs tissue content) and xenograft nude mice implanted with different tumor cell types [13,22,24,41,86,87,88,89]. However, it should be noted that most studies have been performed in experimental settings evaluating the suppression of tumor development and only few investigations have been realized in therapeutic settings, evaluating the capability of PUFAs to eradicate established tumors [24,41,86,87,88,89].

Encouraging results concerning the *in vivo* anti-neoplastic activity of *n-*3 PUFAs have been also obtained from clinical studies, even though they were mainly set-up to investigate cancer prevention and support, rather than cancer therapy [25,30,31,90,91,92,93,94,95,96,97,98,99,100,101]. Indeed, the outcomes mainly investigated included *n-*3 PUFAs membrane incorporation, immune and inflammatory responses, oxidative status, as well as body weight and composition or quality of life [31,96,97,99,100,101]. Few studies addressed *n-*3 PUFAs supplementation and decrease of tumor size or extension of patient survival [90,91,92,93,95,96,98,100] (Table 1).

**Table 1:** Overview of human studies investigating the clinical outcome or prognosis in cancer patients supplemented with *n-*3 polyunsaturated fatty acids (PUFAs).

Cancer Type	Study Type	Enrolled Subjects	Pts (n)	FA/Daily	Objectives	Outcomes	Ref.
CRC	Phase II double-blind RCT	Patients under-going liver resection surgery for CRCLM	43 (T) 45 (C)	EPA (2 g)	To evaluate: ki67 proliferation index; safety and tolerability; tumor FA content; CD31-positive vascularity.	No difference in Ki67 proliferation index. Treatment was safe and well tolerated. EPA was incorporated into CRC liver metastasis tissue. Treatment reduced vascularity of CRC liver metastases. In the first 18 months after CRCLM resection, EPA-treated patients obtained OS benefit compared with control, although early CRC recurrence rates were similar.	[95]
CRC	Systematic review and meta-analysis: 9 trials published until September 2014	Patients with CRC undergoing concomitant surgery (5 trials) or chemotherapy (3 trials)	242 (T) 233(C)	EPA + DHA (2.2 g: median daily dose (range 0.6-4.8)	To evaluate the effects of *n-*3 PUFAs on inflammatory mediators (cytokines and acute phase proteins): IL-6 and IL-1β, TNF, CRP and CRP/albumin ratio.	Benefits on some inflammatory mediators, but they are specific for some supplementation protocols (duration, dose, route) and concomitant anti-cancer treatment: reduction in IL-6 occurs in surgical patients that received 0.2 g/kg of FO parenterally at postoperative period (*p =* 0.002); increase in albumin occurs in surgical patients that received >2.5 g/d of EPA+DHA orally at preoperative period (*p =* 0.038); in patients undergoing chemo- therapy, the supplementation of 0.6 g/d of EPA+DHA during 9 week reduces CRP levels (*p =* 0.017), and CRP/albumin ratio (*p =* 0.016).	[101]
CRC	RCT with two arms, parallelgroups,open label	Patients with advanced CRC never submitted to chemotherapy	17 (T) 13 (C)	FO (2 g); (0.6g/day EPA + DHA)	To evaluate clinical outcomes during and after chemotherapy in individuals with CRC who received FO in the first 9 week of treatment. Outcomes assessed were: number of chemotherapy cycles administered; days undergoing chemotherapy; number of delays and interruptions in the admi-nistration of chemotherapy; number of hospitalizations during chemothery; tumor progression; values of CEA; days until events (death and progression); and 3-year survival.	Time to tumor progression was significantly longer in treated (593 days ±211.5) *vs* control (330 days ±135.1) patients (*P =* 0.04); treated patients presented also lower CEA values after chemotherapy (however these differences were not statistically significant); other outcomes did not differ between groups.	[90]
Breast cancer	Open*-*label, one-arm phase II study	Metastatic breast cancer patients undergoing anthracycline-based chemotherapy (5-FU, epirubicin, cyclophosphamide) at first-line treatment for metastases	25 (T)	DHA (1.8 g)	To investigate the efficacy and safety of adding DHA to an oral supplement ROS generating chemotherapy treatment, by measuring response rate and OS.	No adverse effects. Higher plasma DHA concentrations were associated to greater median time to progression (8.7 months) and OS (34 months) compared to patients with low plasma DHA levels (3.5 and 18 months, respectively).	[91]
Breast cancer	A population*-*based follow-up study (using resources from the Long Island Breast Cancer Study Project)	Women newly diagnosed with first primary in situ (16%) or invasive (84%) breast cancer	1463	Variable dietary fish intake	To investigate whether dietary *n-*3 PUFA intake benefits survival after breast cancer.	All cause mortality was reduced by 16% to 34% among women with breast cancer who reported a high intake of fish and *n-*3 PUFAs.	[100]
NSCLC	Two-arm, non*-*randomized phase II study	Patients with advanced NSCLC undergoing platinum-based chemotherapy (carboplatin with vinorelbine or gemcitabine) as first-line treatment	15 (T) 31 (C)	EPA + DHA (2.5 g)	To evaluate whether the combination of FO and chemotherapy provided a benefit over standard of care on response rate and clinical benefit from chemotherapy.	Plasma EPA and DHA were higher in treated patients (*p* < 0.001 and *p =* 0.004, respectively). Treated patients had an increased response rate and greater clinical benefit compared with the control group (60.0% *vs* 25.8%, *p =* 0.008; 80.0% *vs* 41.9%, *p =* 0.02, respectively). The incidence of dose-limiting toxicity did not differ between groups (*p =* 0.46). One-year survival tended to be greater in treated patients (60.0% *vs* 38.7%; *p =* 0.15).	[93]
NSCLC	Prospective RCT	Adva*-*ced NSCLC receiving paclitaxel and cisplatin/carboplatin treatment	46 (T) 46 (C)	EPA (2 g)	To compare the effect of an oral EPA enriched supplement with an isocaloric diet on nutritional, clinical and inflammatory parameters and health-related quality of life. Response to chemotherapy and survival were also evaluated.	Improvement of energy and protein intake, body composition, and decreased fatigue, loss of appetite and neuropathy. There was no difference in response rate or OS between control and EPA group.	[96]
Pancreatic Cancer	A systematic evaluation of results of 11 prospective cohort RCTs	Unresectable pancreatic cancer patients	602 (T) 765 (C)	EPA (range 1-6 g) and/or DHA (range 0.96-1 g)	To systematically evaluate results of trials examining the effects of *n-*3 PUFA consumption on body weight, lean body mass, resting energy expenditure, and OS.	A significant increase in body weight (*p* < 0.00001) and lean body mass (*p* < 0.00001), a significant decrease in resting energy expenditure (*p =* 0.03), and an increase in OS (130–259 days *vs* 63–130 days) in patients who consumed an oral nutrition supplement enriched with *n-*3 PUFAs compared to those who consumed conventional nutrition.	[98]

Abbreviations: Pts (n), number of patients; FA, fatty acids; C, control; T, treated; CRC, colorectal; CRCLM, colorectal cancer liver metastases; OS, overall survival; RCT, randomized controlled trial; CEA, carcinoembryonic antigen; NSCLC, non*-*small-cell lung cancer; IL, interleukin; TNF, tumor necrosis factor ; CRP, C-reative protein; FO, fish oil; PUFAs, polyunsaturated fatty acids; DHA, docosaexaenoic acid; EPA, eicosapentaenoic acid; 5-FU, 5-fluorouracil; Ref., reference number.

Thus, although in these studies *n-*3 PUFAs supplementation was associated with improvement of clinical outcome and prognosis, the conclusion is limited because of the limited amount of data. 

### 2.2. Triggering of the Intrinsic and Extrinsic Apoptotic Pathways by n-3 PUFAs

Many studies have reported that *n-*3 PUFAs induced apoptosis by triggering the intrinsic mitochondrial and ER pathways. In fact, EPA increased caspase-3 and -9, but not caspase-8, while inducing apoptosis in Ramos lymphoma cells [102]. Different studies in colon cancer (LS-174, HT-29, Caco-2 and COLO 201) cell lines showed that dietary FO [103] or DHA [104,105] modified the expression of Bcl-2 family proteins by increasing the levels of the pro-apoptotic proteins Bak and Bcl-xS and decreasing those of the anti-apoptotic proteins Bcl-2 and Bcl-xL. Similarly, Sun *et al.* [54] observed that DHA induced apoptosis in human Bel-7402 hepatocellular carcinoma cells, by up-regulating caspase-3 and Bax expression levels and downregulating the expression of Bcl-2 and Bim. Recently, Abdi *et al.* [44] demonstrated that EPA and DHA induced apoptosis in myeloma (L363, OPM-1, OPM-2 and U266) cells through mitochondrial perturbation and caspase-3 activation, whereas both compounds did not affect the viability of normal human peripheral blood mononuclear cells. Moreover, the analysis of gene modulation by *n-*3 PUFAs in myeloma cells revealed the modulation of several signal pathways, including nuclear factor (NF)-κB, Notch, Hedgehog, oxidative stress and Wnt, indicating the possible involvement of multiple molecular signals in the initiation of apoptosis by the intrinsic pathway. Finally, the activation of the intrinsic ER stress pathway has been also proposed underlying DHA-induced apoptosis in colon cancer cells. Indeed, Jackobsen *et al.* [106] showed that DHA, while inducing cell death in the aggressive SW620 colon cancer cell line, also induced extensive changes in gene expression patterns (mRNA) of ER stress; they also found abundant presence of phosphorylated eIF2α, increase in cytosolic Ca^2+^ and disturbances in lipid metabolism, suggesting that cytotoxic effects of DHA are associated with signaling pathways involving lipid metabolism and ER stress.

On the other hand, other studies have indicated the activation of the extrinsic pathway in the induction of apoptosis by *n-*3 PUFAs. Indeed, increased expression of both caspase-9 and caspase-8 was reported in EPA- [107] and DHA- [108] induced apoptosis in human HL-60 leukemia and Caco-2 colon cancer cells, respectively. In the case of colon cancer cell apoptosis, tBid expression was also enhanced, indicating a contribution of caspase-8 also to the activation of the mitochondrial pathway. Accordingly, in our laboratory we found the involvement of caspase-8 in DHA-mediated apoptosis in pancreatic and bladder cancer cell lines [65].

Finally, both DHA and EPA could exert an important pro-apoptotic effect in different colorectal cancer (Caco-2, HT-29, HCT116, LoVo, SW480) cells by the downregulation of two key regulatory elements of the extrinsic and intrinsic pathways, FLIP and XIAP, respectively; interestingly, DHA and EPA did not affect the viability of normal human colon mucosal epithelium (NCM460) cells [39].

## 3. Molecules, Signals and Networks Targeted by *n-*3 PUFAs: Upstream Events in the Triggering of the Apoptotic Pathways

Cancer is often described as a disorder of the balance between cell growth and death [5]. On the one hand, defects in signaling pathways promoting cell growth and survival occur in cancer cells and high constitutive levels of MEK/ERK, PI3K/Akt, JAK/STAT or IKK/IκB/NF-κB pathways are frequently observed in human cancers [5,109]. On the other hand, as already mentioned in Section 2, defects along the apoptotic pathways also occur in cancer cells, leading to resistance to apoptosis [5,110]. Therefore, all the molecules, signals and networks involved in cancer cell survival and death are potential targets for apoptosis-based cancer therapies.

The mechanisms by which *n-*3 PUFAs induce apoptosis in tumor cells are not fully determined in molecular terms; however, the proposed main routes of action of *n-*3 PUFAs are: (1) incorporation into cell membranes, leading to changes in the distribution and function of key survival and death signals; (2) generation of lethal levels of intracellular oxidative stress; (3) modulation of eicosanoid metabolites; (4) binding to nuclear receptors, leading to changes in gene expression. These routes may underlie the pleiotropic and multifaceted effects of *n-*3 PUFAs, leading to the induction of apoptosis in cancer cells and/or to the sensibilization of tumor cells to traditional therapies. Therefore, in the context of these four routes of action of *n-*3 PUFAs, in this section we analyze studies investigating the mechanisms underlying the induction of apoptosis, highlighting the potential upstream molecular events targeted by *n-*3 PUFAs to trigger the apoptotic pathways in cancer cells.

### 3.1. Cell Membrane Enrichment in n-3 PUFAs and Changes in the Distribution and Function of Key Survival and Death Signals in Cancer Cells

Once ingested, *n-*3 PUFAs, EPA and DHA are uptaken and incorporated in tumor cell membranes by both passive or carrier-mediated transmembrane translocation [29]. The FA composition of membrane phospholipids can influence multiple cellular functions. It should be noted that DHA, for its high level of unsaturation and presence of several CH-CH_2_-CH repeating units in its molecule, possesses an extremely flexible structure, more flexible than EPA, and it can rapidly isomerize through different conformational states [29]. Therefore, the enrichment of *n-*3 PUFAs in tumor cell membranes and the high molecular disorder originating from their (mainly DHA) incorporation into membrane phospholipids may affect physical-chemical properties of membranes, including their fluidity, permeability, deformability, as well as their lipid microdomain formation [29,111]. Plasma membrane is composed of microdomains of saturated lipids that segregate together to form “lipid rafts”. Lipid rafts are enriched in glycosylphosphatidylinositol-linked proteins, contain several signaling proteins (e.g., epidermal growth factor receptor, EGFR) and play a key role in cell signal transduction, mainly by facilitating the association of signal molecules (e.g., those involved in cell survival). Cholesterol is a critical lipid component for lipid raft integrity and function, and DHA have poor affinity for cholesterol and influences lipid rafts, modifying their biochemical and biophysical features and changing their composition and/or the activity of raft-related signaling molecules. Therefore, concerning the regulation of apoptosis, *n-*3 PUFAs have the potential to modulate the function of death receptors, growth factor receptors, cytokines and hormones receptors, as well as oncogenes, tumor suppressor genes and signal transduction secondary messangers (e.g., adapter proteins, receptor-associated enzymes, protein kinases and phosphatases). As a consequence, *n-*3 PUFAs may alter the activation of transcription factors and expression of genes as well as the phenotype of tumor cells [111]. Thus, cell membrane enrichment in *n-*3 PUFAs can influence multiple cellular functions at multiple biological levels. Moreover, noteworthy, it has been reported that there are significant differences between tumor and normal cells in *n-*3 PUFAs uptake and membrane distribution, being tumor cells deficient in PUFAs (especially in arachidonic acid-ARA, EPA and DHA) as compared to normal cells, since they have decreased activity of Δ^5^ and Δ^6^ desaturases. Although the exact reason for the low activity of desaturases in cancer cells is not known, it has been proposed that it might be a defence mechanism adopted by tumor cells to protect themselves from toxic molecules such as free radicals derived from *n-*3 PUFAs peroxidation in cancer cells (see Section 3.2) [112,113]. Therefore, the specific enrichment of tumor cell membranes with *n-*3 PUFAs, EPA or DHA is one of the possible reasons underlying the capability of *n-*3 PUFAs to induce cytotoxicity in tumor cells, with no or little action on normal cells.

Discoveries over the last decade propose that *n-*3 PUFAs incorporation into cancer cell membranes is essential for apoptosis by *n*3-PUFAs in different cancer cell models [26,113,114,115]. However, currently, the precise mechanism of how a selective change in DHA and EPA content of membranes translates to a change in signaling events to induce apoptosis is not completely clear. Therefore, in the next sections (from Section 3.1.1 to 3.1.6), we analyze the studies investigating this issue. We take into consideration different possible actions by *n-*3 PUFAs, such as the displacement of lipid raft associated onco-proteins as well as the modulation of different survival signaling pathways in tumor cells, including Wnt/β-catenin, MAPK/Erk, PI3K/Akt/mTOR, JAK-STAT and NF-κB pathways.

#### 3.1.1. Changes in Lipid Raft-Associated Onco-Proteins by *n-*3 PUFAs

The involvement of the modulation of EGFR and HER-2 signals in *n-*3 PUFAs-induced apoptosis has been reported by different studies, performed in different types of cancer (mainly breast cancer) cells. In 2007, Schley and coworkers [116] showed that apoptosis induced by a combination of EPA and DHA in MDA-MB-231 breast cancer cells was due to changes in lipid raft composition, leading to a decrease of EGFR levels as well as an increase of EGFR and p38 mitogen*-*activated protein kinase (MAPK) phosphorylation. Accordingly, in oral SCC cells, it was found that DHA- and EPA-induced apoptosis was mediated by amplification of the EGFR/ERK/p90RS kinase (*K*) pathway (*i.e.*, EGFR autophosphorylation, sustained phosphorylation of ERK1/2 and of its downstream target p90RSK); to note, the viability of normal keratinocytes was not affected [42]. In contrast with these results, in three different (A549 lung, WiDr colon and MDA-MB-231 breast) cancer cell models it was found that DHA-induced apoptosis was caused by the exclusion of EGFR from caveolin*-*rich lipid raft fractions, resulting in a decreased association of Ras with Sos1 and the subsequent downregulation of Erk signaling; these data were confirmed *in vivo*, using xenograft athymic mice implanted with A549 cells [117]. Similarly, a reduction of EGFR activation was observed in EPA- or DHA-induced apoptosis in breast cancer (MDA-MB-231 and MCF-7) cells, associated to a reduction of Bcl2 and caspase-8 expression; moreover, DHA (probably related to its better capability to change lipid raft properties), but not EPA, also slightly reduced EGFR concentration [118]. More recently, in line with these results, it was found that DHA had the capability of decreasing cell surface levels of lipid rafts via their internalization and then fusion with lysosomes in MDA-MB-231 breast cancer cells. This implied that DHA displaced several raft-associated onco-proteins, including EGFR, Hsp90, Akt, and Src and also decreased total levels of those proteins via multiple pathways, including the proteasomal and lysosomal pathways, thereby decreasing their activities such as Hsp90 chaperone function [119]. Then, the therapeutic potential of DHA in the treatment of HER-2 positive breast cancers has been reported by two investigators. Ravacci [43], Mason [120] and coworkers showed that DHA induced apoptosis in transformed human mammary epithelial (HB4aC5.2) cells and in breast cancer (BT-474) cells, respectively, by the deplacement of HER-2 from lipid rafts and the decrease of Akt and ERK1/2 activation; no effects were observed in related untransformed (HB4a) cells.

New insight into the potential application of *n-*3 PUFAs in breast cancer treatment was also provided by a recent investigation in MCF-7 and T47D breast cancer cells, showing that DHA and EPA could shift the pro-survival estrogen signal to a pro-apoptotic effect by increasing the G protein coupled estrogen receptor 1-cyclic adenosine monophosphate**-**protein kinase A (GPER1-cAMP-PKA) signaling response, blunting EGFR, Erk 1/2 and AKT activity [121].

It is of interest that it has been also demonstrated that pre-treatment of estrogen receptor negative MDA-MB-231 cells with DHA increased the anti-cancer effects of doxorubicin, by increasing the plasma membrane raft content of CD95 and FADD. [122].

Finally, in prostate cancer (PC3 and LNCaP) cells, growth suppression by DHA was due to changes in cell plasma membrane phospholipid content, leading to the alteration of phosphatidylinositol phosphates (PIPs) content, PI(3,4,5)P_3_ (PIP_3_) and Akt localization, inhibition of Akt phosphorylation and thus of the AKT survival signaling pathway [123].

#### 3.1.2. Inhibition of the Wnt/β-Catenin Pathway by *n-*3 PUFAs

Wnt functions causing an accumulation of β-catenin in the cytoplasm and its eventual translocation into the nucleus, to act as a transcriptional coactivator of transcription factors that belong to the T cell factor/lymphoid enhancer factor (TCF/LEF) family. Dysregulation of Wnt signaling and β-catenin expression is believed to be central in the regulation of tumor cell apoptosis [109].

In 2007, Calviello *et al.* [124] proposed that DHA exerted pro-apoptotic effects in colon cancer cells through proteasomal-dependent degradation of β-catenin, leading to dow*n-*regulation of the expression of TCF-β-catenin target genes such as survivin (a IAP family member). Then, Lim [53,59], Song [125] and coworkers showed that DHA- and EPA-induced apoptosis in human cholangiocarcinoma, hepatocellular and pancreatic carcinoma cells was caused by the inhibition of the β-catenin signaling pathway through two systems of β-catenin degradation, such as the activation (by dephosphorylation) of glycogen synthase kinase-3β (GSK-3β) and the induction of the formation of β-catenin/Axin/GSK-3β binding complex; similar results were obtained *in vivo*, in Fat-1 transgenic mice implanted with mouse pancreatic cancer (PANC02) cells [125]. More recently, the inhibition of the Wnt/β-catenin pathway was also found to be involved in growth suppression of breast MCF-7 cancer cells *in vitro* and in therapy experiments *in vivo*, performed in Babl/c mice bearing 4T1 mouse breast cancer and fed with a 5% FO diet [126].

#### 3.1.3. Modulation of the Mitoge*n-*Activated Protein Kinase (MAPK)/ERK (or Ras/Raf/MEK/ERK) Pathway by *n-*3 PUFAs

The MAPK/ERK pathway includes many MAPK proteins (originally called extracellular signal-regulated kinases, ERK), which function by adding phosphate groups to a neighboring protein, leading to change the expression of genes specific for molecules involved in cell cycle and apoptosis. This pathway represents a necessary step in the development and progression of many cancers. Although the activation of ERK is traditionally linked to cell survival and proliferation, recent studies have demonstrated that this is not always the case and ERK activation can also cause growth arrest or apoptosis [109]. Indeed, as reported below, both activation and inhibition of ERK have been associated with *n-*3 PUFAs-induced tumour cell apoptosis, suggesting that tissue- or cancer-specific mechanisms of *n-*3 PUFAs action might occur.

In 2008, Serini *et al.* [68] showed that DHA-induced apoptosis was due to decreased levels of phosphorylated MAPKs, especially ERK1/2 and p38 in lung cancer cells. Accordingly, *n-*3 PUFAs induced apoptosis in breast cancer cells *in vitro* and in a Fat-1 mice breast cancer model, by inhibition of the MEK/ERK/Bad signaling pathway; the inhibition was induced through the increased expression of the integral membrane protein syndeca*n-*1 (SDC-1) [127]. On the other hand, in gastric cancer cells, DHA-induced apoptosis was caused by the activation of ERK and c-Jun *N-*terminal kinase (JNK), leading to the activation of AP-1 transcription factor, which induced the expression of apoptotic genes [52].

#### 3.1.4. Inhibition of the PI3K/Akt/mTOR Pathway by *n-*3 PUFAs

PI3K (phosphatidylinositol-3-kinase**)** is one of the intracellular pathways responsible for the transmission of anti-apoptotic signals by cell survival factors. Phosphatase and tensin homologue deleted on chromosome ten (PTEN) is a lipid phosphatase, which catalyzes the dephosphorylation of PIP3 and thus serves as a major negative regulator of PI3K/Akt signaling; when it is phosphorylated, it becomes inactive. Akt (or protein kinase B, PKB) is a serine/threonine kinase, activated in response to cytokines and growth factors through its translocation to the plasma membrane and its phosphorylation at two key residues (Thr308 and Ser473). Akt activation promotes directly cell survival and protect cells from apoptosis by inactivating components of the cell death machinery (e.g., caspase-9, Bad); in addition, Akt promotes indirectly cell survival and protect cells from apoptosis by activating transcription factors such as NF-κB, that induces the transcription of pro-survival and anti-apoptotic genes. Mammalian target of rapamycin **(**mTOR) is a protein kinase that integrates both intracellular and extracellular signals, and serves as a central regulator of cell metabolism, growth and survival. It regulates the activity of p70S6K and eukaryotic initiation factor (eIF)4E binding protei*n-*1 (4E-BP1). The mTOR pathway is deregulated and activated in several types of cancer, significantly contributing to the enhancement of proliferation and the inhibition of autophagy; overexpression of downstream mTOR effectors 4E-BP1, S6K and eIF4E4 leads to poor cancer prognosis. Therefore, inhibition of mTOR activity disrupts the balance between pro- and anti-apoptotic proteins, enhancing tumor cell death [109].

It was shown that EPA and DHA induced apoptosis in MDA-MB-231 breast cancer cells *in vitro* [128,129] and in a xenograft animal model [129] by the inhibition of the survival Akt/NF-κB signaling pathway, due to the inactivation of PI3K, through increased PTEN expression by *n-*3 PUFAs. On the other hand, DHA-mediated apoptosis in colon cancer (Caco-2) cells, was due to the inactivation of PI3K induced by reduced PTEN phosphorylation by *n-*3 PUFAs. This inactivation promoted inhibition of Akt/PKB and thus of Bad and forkhead transcription factor (FKHR); to note, the viability of normal colon (NCM460) cells was not compromised [40,130]. Then, the suppression of the activity of (3’-phosphoinositide-dependent kinase 1)-PDK1/Akt/Bad signaling was demonstrated underlying *n-*3 PUFA-induced apoptosis in prostate cancer (PC3, LNCaP and DU145) cells *in vitro* and *in vivo*; moreover, the suppression was dependent on the upregulation of SDC-1, and 15-LOX-1-mediated metabolism of DHA was required for SDC-1 upregulation [64].

From recent studies, it has emerged that DHA can also simultaneously induce apoptosis and autophagy in cancer cells, and this process involves mTOR repression [131,132]. Indeed, DHA treatment in human cervical cancer cells led to autophagy via p53-mediated (AMP-activated protein kinase)-AMPK/mTOR signaling (*i.e.*, mTOR inhibition and AMPK activation), and DHA-induced autophagy sensitized tumor cells to apoptosis [131]. Then, in no*n-*small cell lung cancer cells, it was shown that DHA-induced apoptosis and autophagy were associated to mTOR suppression induced by both AMPK activation and PI3K/Akt inhibition; these data were confirmed in Fat-1 transgenic mice implanted with Lewis lung cancer cells [133].

#### 3.1.5. Inhibition of the JAK-STAT Pathway by *n-*3 PUFAs

The JAK-STAT system consists of a receptor (activated by interferons, interleukins, growth factors, or other chemical messengers), the Janus kinase (JAK) and the signal transducer and activator of transcription (STAT) proteins. STAT proteins once activated translocate into the nucleus, where they bind to DNA, promoting the transcription of specific genes affecting basic cell functions such as cell growth and death. The activation of STAT3 pathway in tumor cells is mainly due to the effect of tumor released factors and plays a critical role in tumor cell-survival and chemo-resistance [109].

A very recent work by Rescigno *et al.* [134] demonstrated that DHA-induced apoptosis in aggressive SK-BR-3 breast cancer cells reduced both ERK1/2 and STAT3 phosphorylation; interestingly, DHA only arrested cell cycle progression of no*n-*tumor MCF-10A breast cells, activating p21^Waf1/Cip1^ and p53. Moreover, it was also shown that the elimination of aldehyde dehydrogenase positive cells and the inhibition of mammosphere formation of TICs in human triple negative breast cancer cells by DHA was due to the Src homology region 2 domai*n-*containing protein tyrosine phosphatase-1 (SHP-1)-dependent suppression of STAT3 activation and of its downstream mediators c-Myc and cyclin D1 [85].

#### 3.1.6. Inhibition of the NF-κB Pathway by *n-*3 PUFAs

NF-κB transcription factor plays a key role in many physiological processes, including inflammation, cell proliferation and death. The aberrant regulation of NF-kB and signaling pathways that control its activity are heavily implicated in promoting pro-survival signaling and may be critical for resistance to chronic oxidative stress (*i.e.*, drug resistance) [2].

In prostate cancer (LNCaP, DU145, PC3) cells, it was shown that DHA synergistically enhanced the cytotoxic effect of docetaxel, through increased apoptosis by suppression of genes involved in the NF-κB pathway [135]. Then, always in prostate cancer (LNCaP and PacMetUT1) cells, it was reported that exposure of cells to DHA attenuated H_2_O_2_-induced NF-κB transcriptional activity and diminished the expression of the downstream anti-apoptotic target survivin; this activity was specific, since it was not observed in normal human prostate (PrEC) cells [136].

### 3.2. Cell Membrane Enrichment in n-3 PUFAs and Increased Oxidative Stress in Tumor Cells

One of the main characteristics of *n-*3 PUFAs is the fact that they are optimal substrates for oxidants inside the cell, undergoing thus nonenzymatic lipid peroxidation into cell membranes; moreover, nonenzymatic lipid peroxidation triggers a further increase of the formation of oxygen radicals and ROS [29]. Tumor cells contain higher levels of ROS compared to normal cells, principally due to their accelerated metabolism needed to maintain their high proliferation rate. Thus, ROS in tumor cells can react with intracellular *n-*3 PUFAs giving rise to nonenzymatic lipid peroxidation products that are highly toxic [38]. The methylene group, located between two double bonds (–CH=CH–CH_2_–CH=CH–), is particularly vulnerable to radical attack by reactive species, thus entailing the abstraction of hydrogen [29]. Moreover, DHA, possessing an additional double bond with respect to EPA, is more susceptible to nonenzymatic lipid peroxidation, providing a variety of lipid hydroxiperoxides and aldehydic breakdown products such as malonaldehyde (MDA; a marker for lipid peroxidation) with toxic as well as prooxidant properties [13,24]. Indeed, the nonenzymatic lipid peroxidation triggers a further increase of the generation of intracellular radical species; the further increase of intracellular ROS levels and oxidative stress in tumor cells by *n-*3 PUFAs (EPA > DHA) causes the disruption of the mitochondrial membrane potential, the release of cytochrome C and thus the triggering of the intrinsic apoptotic pathway (see Section 2). Moreover, DHA can be readly incorporated in mitochondrial membranes, altering their permeability and decreasing the mitochondrial membrane potential [29]. It has been also reported that DHA is mostly present in the mitochondrion in association with cardiolipins; cardiolipin*-*DHA molecules are under attack of radical species, with the consequent decrease of their binding affinity for cytochrome C, enhancement of its release and the release of other pro-apoptotic factors (e.g., Smac/Diablo) from mitochondria to cytosol, and the triggering of the intrinsic apoptotic pathway [29]. In addition, it is known that ROS can also oxidize and inhibit key signaling pathways involved in cell proliferation, survival and apoptosis, such as MAPK and NF-κB pathways [55,137]. Therefore, all these considerations indicate that lipid peroxidation and increased ROS levels play a key role in the induction of tumor cell apoptosis by *n-*3 PUFAs. Interestingly, it has been reported that there are significant differences in tumor *vs* normal cells not only in the uptake and distribution of *n-*3 PUFAs, but also in the ability to generate reactive species and oxidative stress from intracellular *n-*3 PUFAs. Indeed, as mentioned, tumor cells contain higher levels of oxygen radicals compared to normal cells and in presence of DHA they increase the production of cytotoxic lipid hydroperoxydes and other peroxides, undergoing apoptosis. In contrast to tumor cells, normal cells can use DHA to protect themselves from oxidative stress-induced apoptosis through a certain number of mechanisms, including the activation of the survival PI3K/Akt pathway as well as the increased production of cytoprotective molecules such as resolvins and protectins (see also Section 3.3) [138,139,140]. As reported in the next section, several investigators have shown that, as exogenous *n-*3 PUFAs are provided to cancer cells, these FAs can induce apoptosis by augmenting free radical generation and lipid peroxidation, whereas normal cells are not influenced [36,130,138].

Since ROS have been proposed as common mediators of apoptosis, the majority of cytotoxic anticancer agents (including ionizing radiations, most chemotherapeutic agents and some targeted therapies) work through ROS generation [29]. However, although they initially generate ROS production, most cancer cells following prolonged treatment with these drugs develop the capability to reduce ROS levels, resulting in drug-resistance. Evidence exists on the capability of *n-*3 PUFAs to increase both the efficacy of conventional anticancer therapies towards drug resistance and their tolerability towards normal cell damage. Indeed, *n-*3 PUFAs can increase the susceptibility of tumor cells to oxidative stress induced by conventional therapies, by maintaining high ROS levels in cancer cells, thereby precluding drug resistance [27,29]. Moreover, *n-*3 PUFAs can increase the tolerability to conventional therapies, by promoting both the selective induction of letal levels of oxidative stress in tumor cells and the selective production of protective lipid mediators in normal cells. Both activities have important therapeutic potential, further supporting the use of *n-*3 PUFAs as adjuvant in conventional cancer therapies.

#### Increased Oxidative Stress in Cancer Cells by *n-*3 PUFAs and Induction of Apoptosis

Early *in vitro* studies performed in breast [60] and pancreatic [56] cancer cells proposed the involvement of oxidative mechanisms in the induction of cancer cell apoptosis by EPA and *n-*3 PUFAs, respectively; interestingly, increased oxidative stress and apoptosis were not observed in human normal cells, such as fibroblasts [60]. Further studies have shown that oxidative stress in cancer cells was generated by *n-*3 PUFAs through both generating lethal ROS levels and decreasing anti-oxidant activities in tumor cells [38].

In our laboratory, we showed that DHA promoted apoptosis in the human PaCa-44 pancreatic cell line through the induction of an active extrusion process of intracellular reduced glutathione (GSH), depleting tumor cells of one of the endogenous antioxidant defences, and increasing thus tumor cell sensibility to lipid peroxidation and oxidative stress [57]. This data has important implications for cancer therapy, since elevated GSH levels in tumors have been associated with resistance to apoptosis and chemotherapy [29]. Similarly, Ding and co-workers [141] found downregulation of the antioxidant enzyme superoxide dismutase 1 (SOD1) expression in the DHL-4 lymphoid cell line undergoing apoptotsis by DHA. Then, in the same laboratory, it was shown that DHA-mediated cytotoxicity in human ovarian cancer cell lines was associated to a reduction of glutathione peroxidase (GPx)-4 protein expression and that DHA-mediated cytotoxicity was reversed by vitamin E, suggesting that GPx-4 downregulation was due to oxidative stress [142]. Moreover, it was reported that the *in vitro* and *in vivo* sensitization of MDA-MB-231 breast cancer cells to anthracyclines (doxorubicin) by DHA was caused by a decrease of cytosolic GPx-1 activity and a concomitant increase of ROS levels [143].

On the other hand, *n-*3 PUFAs can also promote apoptosis by increasing lipid peroxidation and intracellular oxidative stress. It has been shown that DHA enhanced arsenic-trioxide-induced apoptosis in arsenic-trioxide resistant HL-60 (myeloid leukemia), SH-1 (hairy cell-leukemia), and Daudi (Burkitt lymphoma) cell lines by an increase of lipid peroxidation and a reduction of the mitochondrial membrane potential; these effects were reversed by the addition of the antioxidant vitamin E [144]. Similarly, Lindskog *et al.* [66] showed that DHA-mediated neuroblastoma cell death was associated with production of ROS and depolarization of the mitochondrial membrane potential, whereas vitamin E inhibited both mitochondrial depolarization and cell death; of note, nontransformed fibroblasts were not substantially affected by DHA. Moreover, DHA also significantly enhanced the cytotoxicity of arsenic trioxide, nonsteroidal antiinflammatory drug (diclofenac) and conventional chemotherapeutic agents (cisplatin, doxorubicin and irinotecan) both in chemosensitive and in multidrug-resistant neuroblastoma cells. More recently, similar effects were found in human HT-29 colorectal adenocarcinoma cells treated with DHA-combined treatment with 5-FU, OX and irinotecan (IRI); the anticancer action of DHA, observed in presence of low doses of chemotherapeutic drugs (1 μM 5-FU, 1 μM OX and 10 μM IRI), was carried out by loss of mitochondrial membrane potential and caspase-9 activation [145]. Increased lipid peroxidation associated to the activation of the intrinsic apoptotic pathway was confirmed by other investigators as mechanism underlying DHA- or EPA-mediated apoptosis, in different human cancer colon (HT-29 and Caco-2) [146] and gastric (MGC and SGC) [147] cell lines. In addition, in DHA-induced apoptotic human papillomavirus (HPV)-infected cancer cells, it was reported that the overproduction of mitochondrial ROS by DHA promoted the activation of the cellular ubiquiti*n-*proteasome system, which leads to the degradation of E6/E7 oncoproteins, essential in the maintenance of HPV-associated malignancies [148].

Furthermore, it has been found that oxidative stress could induce apoptosis by triggering not only the intrinsic pathway, but also by the extrinsic pathway. Indeed, Kang *et al.* [149] found that DHA promoted apoptosis in MCF-7 breast cancer cells *in vitro* and *in vivo* via both ROS formation and caspase-8 activation, in that antioxidants or knockdown of caspase-8 each effectively abrogated cytotoxicity by DHA. To explain caspase-8 activation, the authors have hypothesized that ROS accumulation in plasma membrane lipid rafts might induce the assembly of DISC, triggering thus the extrinsic pathway. Then, the same investigators [58] also showed the induction of both ROS accumulation and caspase-8-dependent cell death by EPA and DHA, in human pancreatic cancer (MIA-PaCa-2 and Capa*n-*2) cells *in vitro* and in xenografts athymic nude mice fed with 5% FO.

Recently, Jeong *et al.* [137] reported that the activation of MAPKs such as ERK/JNK/p38 was involved in DHA-induced apoptosis and that this activation was associated with mitochondrial ROS overproduction. Accordingly, Zhang *et al.* [55] showed that EPA caused apoptosis in HepG2 cells by evoking ROS formation, leading to both [Ca2+] accumulation and increased activation of JNK; both events promoted MOMP, the release of cytochrome C from mitochondria, and the activation of caspase-9 and caspase-3; to notice, EPA had no significant effect on the viability of normal liver (L-02) cells.

Finally, while it is well established that excessive ROS can instigate apoptosis, emerging data have also revealed a signaling role for ROS in the activation of autophagy. In PC3 and DU145 prostate cancer cells, with mutant p53 and exposed to DHA, it was found that ROS-mediated apoptosis and autophagy were caused by the inhibition of Akt-mTOR signaling [132]. According to these results, Zajdel *et al.* [150] showed that oxidative stress induced in human A549 lung cancer cells by EPA and DHA influenced apoptosis as well as tumor cell autophagy; the inhibition of the autophagic process suppressed cell death and decreased activation of caspase-3/7, indicating that EPA- and DHA-mediated autophagy could amplify cancer cell apoptosis.

### 3.3. Cell Membrane Enrichment in n-3 PUFAs and Changes in the Level and Quality of Eicosanoid Metabolites

Eicosanoids are generally considered as oxidized derivatives of 20-carbon FAs in the cell membrane, such as ARA (20:4*n-*6) and EPA. They include prostaglandins (PGs), thromboxanes (TXs), leukotrienes (LXs) and lipoxins (LXs). The major *n-*6 PUFA ARA, because of its prevalence in the phospholipids of cell membranes, is generally the major substrate for eicosanoid synthesis. Once released from membrane phospholipids, free ARA acts as a substrate for cyclooxygenases (COXs), lipoxygenases (LOXs) and cytochrome P450 enzymes; COX enzymes lead to 2-series PGs (e.g., PGE2) and TXs, and LOX enzymes to 4-series LTs and LXs, known as pro-inflammatory and pro-tumorigenic mediators. In fact, inflammation confers to tumor survival and drug resistance. On the other hand, EPA is also a substrate for COXs, LOXs and cytochrome P450 enzymes, giving rise to 3-series PGs (e.g., PGE3) and TXs and to 5-series LTs, known as anti-inflammatory and anti-tumorigenic mediators. These bioproducts bind specific receptors, usually G protein*-*coupled receptors, leading to the activation of signaling pathways involved in the regulation of cancer cell growth and death [9,13]. In addition, EPA and DHA give rise to anti-inflammatory and inflammation resolving metabolites, including resolvins produced from EPA (E-series) and DHA (D-series) and protectins and maresins produced from DHA [113,139,140,151,152]. Anti-inflammatory LXs, resolvins and protectins inhibit the expression of pro-inflammatory cytokines and adhesion molecules, thereby inhibiting tumor cell growth and invasion. Moreover, as mentioned in Section 3.2, it has been proposed that they behave as endogenous cytoprotective molecules for normal cells against lipid peroxidatio*n-*mediated damage by *n-*3 PUFAs. Indeed, enrichment of normal cell membranes in EPA and DHA, both *in vitro* and *in vivo,* may allow normal cells to produce enhanced amounts of resolvins and protectins, protecting themselves against toxic chemicals such as anti-cancer drugs [81]. Tumor cell membrane enrichment in *n-*3 PUFAs can induce changes in the level and quality of eicosanoid products by two main ways: (1) directly, by increasing specific metabolites derived from their metabolic conversion (e.g., PGE3); (2) indirectly, by inhibiting the conversion of ARA to pro-tumorigenic *n-*6 series eicosanoids (e.g., PGE-2). This second way might be pursued by displacing ARA from cell membranes (*i.e.*, *n-*3 PUFAs membrane incorporation partially replace ARA, reducing its availability), by competing with ARA for enzymes (e.g., EPA can act as an alternative substrate for COX-2, leading to a reduction in PGE2 in favour of PGE3), or by inhibiting NF-κB activation, thus decreasing COX-2 enzyme expression [9,29]. Moreover, DHA can inhibit COX-2 activity by binding the substrate channel of COX-2 [9].

COX-2 is overexpressed in many types of cancer leading to the formation of excess of PGE2 [153], and the autocrine COX-2/PGE2 pathway can confer tumor cell resistance to apoptosis by different ways, including the up-regulation of the β-catenin and Ras/Raf/MEK/ERK signaling pathways [154].

#### Modulation of Eicosanoid Bioproducts by *n-*3 PUFAs and Induction of Cancer Cell Apoptosis 

Several studies have indicated that the modulation of eicosanoid production by *n-*3 PUFAs (mainly EPA) may contribute to the induction of apoptosis in cancer cells.

Some investigations have demonstrated that *n-*3 PUFAs can inhibit the autocrine anti-apoptotic COX-2/PGE2 pathway in tumor cells, leading thus to cancer cell apoptosis. Early *in vitro* and *in vivo* studies reported that decreased PGE2 production was associated with decreased growth of prostate [155,156] and breast [157] cancer cells. Furthermore, *n-*3 PUFAs inhibited tumor cell growth in a xenograft prostate cancer model by decreasing PGE2 as well as COX-2 levels [158]. Later, Funahashi *et al.* [159] showed that EPA decreased the growth of COX-2-positive and COX-2-negative PaCa pancreatic cancer cells and the COX-2-dependent mechanism was mediated by the binding of PGE3 to EP2 and EP4 receptors. Accordingly, dietary intake of *n-*3 PUFAs decreased the pancreatic cancer cell growth in a xenograft model through increasing PGE3 and decreasing PGE2 in tumor tissues. The dow*n-*regulation of COX-2 by *n-*3 PUFAs might be a crucial mechanism underlying their apoptotic effect in other types of tumors, including colon cancer [160,161]. In colorectal cancer cells, it was shown that EPA not only decreased COX-2 expression and PGE2 formation, but also increased the COX-dependent formation of EPA-derived metabolites [153]. All these results suggest that EPA may act as a “natural COX inhibitor”. Very recently, Zhang C. *et al.* [113] found that the tumoricidal action of *n-*3 PUFAs on LoVo and RKO colorectal cancer cells *in vitro* was associated not only with the decreased production of pro-inflammatory PGE2 and LTB4, COX-2, arachidonate 5-LOX and microsomal PGE synthase expression, but also with the increased formation of anti-inflammtory LXA4, supporting the hypothesis that LXs, resolvins and protectins have a direct growth inhibitory action on tumor cells; in contrast, 5-FU produced opposite effects on these indices. On the other hand, concerning DHA metabolites, Gleissman *et al.* [138] showed that the cytotoxic action exerted by DHA in neuroblastoma cells was related to its conversion by 15-LOX and, at much lower degree by autoxidation, to 17-hydroxydocosahexaenoic acid (17-HDHA), via 17-hydroxyperoxydocosahexaenoic acid (17-HPDHA), a compound with significant cytotoxicity potency compared to DHA. In normal nervous tissue, DHA was converted by 5-LOX to anti-inflammatory and cytoprotective resolvins and protectins. In contrast, although neuroblastoma cells contained both 15-LOX and 5-LOX enzymes, the complete conversion of DHA into resolvins and protectins did not take place in cancer cells; thus, 17-HPDHA accumulated and exerted high cytotoxicity. Moreover, DHA, similarly to EPA, inhibited the secretion of PGE2 and augmented the cytotoxic potency of the COX-2-inhibitor celecoxib, by competing with ARA metabolites and by binding to catalytic sites of elongases, desaturases, and COX-2.

### 3.4. Binding of Nuclear Receptors by n-3 PUFAs and Changes in Gene Expression

Once released from the cell membrane, *n-*3 PUFAs can bind nuclear receptors such as peroxisome proliferator activating receptors (PPARs) in tumor cells [26], which, as ligand-activated transcription factors, regulate the expression of specific/target genes involved in several biological processes, including lipid metabolism and cell death. However, many of nuclear receptor-mediated effects of EPA and DHA are still unexplored.

It was shown that DHA-induced apoptosis in Reh and Ramos cells was mediated by PPARγ, which in turn up-regulated the p53 protein, leading to the activation of caspase-9 and caspase-3 [77]. Moreover, *in vitro* treatment of breast [162] and prostate [163] cancer cells with DHA activated PPARγ, which in turn up-regulated SDC-1 expression, inducing thus apoptosis. According to these results, O’Flaherty [164], Hu [165] and coworkers showed that 15-LOX metabolites of DHA, such as 17-HPDHA, 17-HDHA, 10,17-dihydroxy- and 7,17-dihydroxy-DHA, while exerting a more potent cytotoxicity on prostate PC3 cancer cells than DHA, like DHA induced apoptotic PC3 cells to activate a PPARγ reporter, which up-regulated SDC-1 expression; apoptosis was reduced by pharmacological inhibition or knockdown of PPARγ or SDC-1. In addition, 15-LOX-1-mediated metabolism of DHA was required to upregulate SDC-1 and to regulate the PDK/Akt signaling pathway that elicited prostate cancer cell apoptosis.

**Table 2:** Overview of studies investigating the apoptotic targets of *n-*3 PUFAs in human tumor cell lines *in vitro*.

Cell Lines	Cancer Type	Fatty Acid	Anti-Cancer Drug	Molecular Targets	Ref.
Caco-2, HT-29	Colorectal	FO	-	↓COX-2 signaling:↓Bcl-2 expression	[103]
COLO 201	Colorectal	DHA	-	Bcl-2 family proteins:↑Bak and Bcl-xS;↓Bcl-xL and Bcl-2	[104]
LS-174, Colo 320 (p53-wild-type), HT-29 and Colo 205 (p53-mutant)	Colorectal	DHA	↑Susceptibility to 5-FU	Bcl-2 family proteins:↓Bcl-xL and Bcl-2	[105]
SW620	Colorectal	DHA	-	↑ER stress genes (ERK-ATF4-CHOP pathway); ↑eIF2α, ↑cytosolic Ca^2+^; Bcl-2 family proteins: ↑Bid; ↓Bad and Bik	[106]
Caco-2	Colorectal	DHA	-	Modulation of apoptotic genes: caspase-9 and -8 activation; pro-apoptotic Bcl-2 family, PG family, LOX, PPARα and γ	[108]
Caco-2, HT-29, HCT116, LoVo, SW480	Colorectal	DHA, EPA	-	↓FLIP, ↓XIAP	[39]
SW480, HCT116	Colorectal	DHA	-	↑Proteosomal degradation of β-catenin: ↓TCF-β-catenin target genes expression (survivin)	[124]
Caco-2	Colorectal	DHA	-	↓PI3K and↓p38 MAPK/Akt pathway	[130], [40]
HT-29	Colorectal	DHA	↑ Susceptibility to 5-FU, OX and irinotecan	Caspase-9 activation	[145]
HT-29, Caco-2	Colorectal	EPA, DHA	-	↑Lipid peroxidation, ↓Bcl-2 levels	[146]
HCA-7	Colorectal	EPA	-	↑COX-2-dependent PGE_2_/PGE_3_ switch	[153]
LoVo	Colorectal	EPA^(1)^, DHA^(2)^	-	^(1)^↓PGE2, LTB4, COX-2, ALOX and mPGEs; ^(2)^↑LXA4, ↓LTB4, COX-2, ALOX5 and mPGES; ↑PGE2 and LXA4	[113]
MDA-MB-231	Breast	*n-*3 PUFAs	-	Lipid raft composition: ↑EGFR onco-protein; ↑EGFR and p38 MAPK signaling	[116]
A549, WiDr, MDA-MB-231	Lung, Colorectal, Breast	DHA	-	Lipid raft composition: ↓EGFR onco-protein; ↓EGFR and ERK signaling	[117]
MDA-MB-231, MCF-7	Breast	EPA, DHA	-	↓EGFR signaling; ↓Bcl-2; caspase-8 activation	[118]
MDA-MB-231	Breast	DHA	-	Lipid raft internalization: ↓lipid-raft-associated onco-proteins (EGFR, Hsp90, Akt, Src)	[119]
HB4aC5.2	Breast	EPA	-	Lipid raft diruption : ↓HER-2 onco-protei*n-*mediated Akt and ERK1/2 signaling	[43]
BT-474	Breast	DHA	-	↓HER-2 onco-protei*n-*mediated Akt and ERK1/2 signaling	[120]
MCF-7, T47D	Breast	DHA, EPA	-	↑Estroge*n-*mediated GPER1-cAMP-PKA signaling	[121]
MDA-MB-231	Breast	DHA	↑Susceptibility to doxorubicin	↑CD95-induced apoptosis	[122]
MCF-7	Breast	DHA	-	↓Wnt/β-catenin pathway	[126]
MCF-7, SK-BR-3	Breast	DHA	-	↑SDC-1 expression: ↓MEK/ERK/Bad signaling	[127]
MDA-MB-231	Breast	*n-*3 PUFAs	-	↓PIK3/Akt/NF-κB signaling	[128]
MDA-MB-231	Breast	DHA, EPA	-	↑PTEN: ↓PIK3/Akt/NF-κB signaling and ↓transcription of Bcl-2 and Bcl-XL genes	[129]
SK-BR-3	Breast	DHA	-	↓ERK1/2 and STAT3 signaling	[134]
TIC	Breast	DHA	-	↑SHP-1: ↓STAT3 phosphorylation	[85]
MDA-MB-231	Breast	DHA	↑Susceptibility to doxorubicin	↓GPx-1	[143]
MCF-7	Breast	DHA	-	↑ROS production and capspase-8 activation	[149]
MCF-7	Breast	DHA	-	PPARγ activation: ↑SDC-1 expression	[162]
PC3, LNCaP	Prostate	DHA	-	↓PIP3 and Akt localization: ↓Akt signaling	[123]
PC3, LNCaP, DU145	Prostate	DHA	-	↑SDC-1 expression: ↓PDK1/Akt/Bad signaling	[64]
PC3, DU145	Prostate	DHA	-	↑Mitochondrial ROS: ↓Akt-mTOR signaling	[132]
LNCaP, DU145, PC3	Prostate	DHA	↑Susceptibility to docetaxel	↓NF-κB pathway	[135]
LNCaP, PacMetUT1	Prostate	DHA	-	↓NF-κB pathway: ↓survivin and ↑oxidative stress	[136]
PC3	Prostate	DHA	-	DHA oxidation and 17-HPDHA: binds PPARγ and ↑SDC-1 expression	[163], [164], [165]
A549, BEN	Lung	DHA	-	↑MPK-1:↓ERK1/2 and p38 MAPK phosphorylation	[68]
A549, H1299	Lung	DHA	-	↑AMPK and ↓PI3K/Akt signaling: ↓mTOR	[133]
A549	Lung	DHA, EPA	-	↑Oxidative stress: ↑autophagy	[150]
AGS	Gastric	DHA	-	↑ERK and JNK signaling: ↑AP-1, which induces apoptotic genes expression	[52]
MGC, SGC	Gastric	EPA, DHA	-	↑Lipid peroxidation	[147]
PaCa-44, MIA-PaCa-2, Capa*n-*2	Pancreatic	DHA	-	↑GSH extrusion	[57]
MIA-PaCa-2, Capa*n-*2	Pancreatic	EPA	-	↑ROS production and caspase-8 activation; ↑autophagy	[58]
SW1990, PANC-1	Pancreatic	DHA, EPA	-	↑β-catenin/Axin/GSK-3βcomplex-mediated β-catenin degradation	[125]
PaCa-44, EJ	Pancreatic, Bladder	DHA	-	Caspase-8 activation	[65]
Hep3B, Huh-7, HepG2	Hepatic	DHA, EPADHA	-	↑GSK-3β-mediated β-catenin degradation; ↓COX-2/PGE2 signaling	[53]
Bel-7402	Hepatic	DHA	-	Bcl-2 family proteins: ↓Bcl-2 and Bim;↑Bax; caspase-3 activation	[54]
HepG2	Hepatic	EPA	-	↑ROS-Ca^2+^-JNK mitochondrial pathway	[55]
SCC-13, SCC-25	Oral squamous cell	EPA	-	↑EGFR/ERK/p90RSK signaling	[42]
CCLP1, HuCCT1, SG231	Cholangiocarcinoma	DHA, EPA	-	↓Wnt/β-catenin; ↓COX-2 signaling	[59]
SK-*N-*DZ, SH-SY5Y (chemo-sensitive), SK-*N-*BE(2) (multi-drug resistant), SK-*N-*AS, IMR-32	Neuroblastoma	DHA	↑Susceptibility to cisplatin, doxorubicin and irinotecan	↑ROS production and depolarization of mitochondrial membrane potential	[66]
SK-*N-*BE(2) (multi-drug resistant), SH-SY5Y	Neuroblastoma	DHA	↑Susceptibility to celecoxib	DHA oxidation by 15-LOX to 17-HPDHA; no DHA oxidation by 5-LOX into resolvins and protectins; ↓COX-2/PGE2 signaling	[138]
HeLa (expressing HPV-18), SiHa	Cervical	DHA	-	↑Mitochondrial ROS: ubiquiti*n-*proteasome system activation, leading to E6/E7 onco-proteins degradation	[148]
HL-60	Myeloid leukemia	EPA	-	Caspase-9 and -8 activation	[107]
HL-60 (arsenic trioxide resistant), SH-1, Daudi	Myeloid leukemia, Hairy cell leukemia, Burkitt lymphoma	DHA	↑Susceptibility to arsenic-trioxide	↑Lipid peroxidation	[144]
Ramos	Burkitt’s lymphoma	EPA	-	Caspase-9 and -3 (but not caspase-8) activation	[102]
DHL-4	B cell lymphoma	DHA	-	↓SOD1 expression	[141]
Reh	Acute lymphocytic leukemia	DHA	-	PPARγ activation: ↑p53 protein, activating caspase-9 and -3	[77]
L363, OPM-1, OPM-2, U266	Multiple myeloma	EPA, DHA	↑Susceptibility to bortezomib	↓NF-κB: ↑mytocondrial oxidative stress and caspase-3 activation	[44]
SiHa, A549, MCF-7	Cervical, Lung, Breast	DHA	-	↓p53/AMPK/mTOR signaling: ↑autophagy	[131]
A2780, A2780/CP70, HL-60, Raji, CEM, MCF-7, MM1.S, MM1.R, C8161, HT29, Panc-1	Ovarian, Leukemia, Breast, Multiple myeloma, Colorectal, Pancreatic	DHA	-	↓GPx-4	[142]
PA-1, H1299, SiHa, D54MG	Ovarian, Lung, Cervical, Glioblastoma	DHA	-	↑Mitochondrial ROS: ↑ERK/JNK/p38 signaling	[137]

Abbreviations: EPA, eicosapentaenoic acid; DHA, docosahexaenoic acid; FO, fish oil; PUFAs, polyunsatured fatty acids; 5-FU, 5-fluorouracil; OX, oxaliplatin; COX-2, cyclooxygenase-2; Bcl, B-cell lymphoma; ATF, activating transcription factor; eIF, eukaryotic initiation factor; MAPK, mitoge*n-*activated protein kinase; PG, prostaglandins; PPAR, peroxisome proliferator-activated receptor; LOX, lipoxygenase; ALOX, arachidonate-lipoxygenase; mPGES, microsomal PG synthase; FLIP, FLICE-like inhibitory protein; XIAP, X-linked inhibitor of apoptosis protein; TCF, T-cell factor; PI3K, phosphoinositide 3-kinase; LTB4, leukotriene B4; LX, lipoxin; EGFR, epidermal growth factor receptor; HSP, heat shock protein; GPER, G protei*n-*coupled estrogen receptor; cAMP, cyclic adenosine monophosphate; PKA, protein kinase A; STAT, signal transducer and activator of transcription; PIP3, phosphatidylinositol (3,4,5)-trisphosphate; SDC-1, syndeca*n-*1; MEK, mitogen/extracellular signal-regulated kinase; NF-κB, nuclear factor-κB; PTEN, phosphatase and tensin homolog deleted on chromosome ten; mTOR, mammalian target of rapamycin; JNK, Jun *N-*terminal kinase; GSH, glutathione; ROS, reactive oxygen species; PDK, phosphoinositide-dependent kinase; ERK, extracellular-signal-regulated kinase; GSK-3β, glycogen synthase kinase-3β; p90RSK, 90 kDa ribosomal protein S6 kinase ; SOD-1, superoxide dismutase-1; 17 HPDHA, 17-hydroxyperoxydocosahexaenoic acid; AMPK, AMP-activated protein kinase; GPx, glutathione peroxidase; Ref., reference number.

**Table 3:** Overview of studies investigating apoptotic targets involved in the suppression of tumor growth by *n-*3 PUFAs in animal models.

Animal Model	Cancer Type	Diet Fatty Acid	Anti-Cancer Drug	Molecular Targets	Ref.
Athymic nude mice implanted with human tumor xenograft HCT-15	Colorectal	FO	-	↓COX2, HIF-1α/VEGF-A and MMPs signal pathways	[166]
Babl/c mice bearing 4T1 mouse breast cancer	Breast	FO	-	↓Wnt/β-catenin pathway	[126]
Athymic nude mice implanted with human tumor xenograft MDA-MB-231	Breast	FO	-	↑PTEN expression: ↓PIK3/Akt/NF-κB signaling, ↓transcription of Bcl-2 and Bcl-XL genes, ↑caspase-3 activation	[129]
Spontaneous NMU-induced rat mammary tumor	Breast	FO	↑Susceptibi-lity to epirubicin	↓GPx-1 response	[143]
Athymic nude mice implanted with human tumor xenograft MCF-7	Breast	FO	-	↑ROS production and caspase-8 activation	[149]
Athymic nude mice implanted with human tumor xenograft MDA-MB-231	Breast	EPA or DHA ethyl esters	-	↓PGE2 production	[157]
Athymic nude mice implanted with human tumor xenograft DU145	Prostate	FO	-	↓PGE2 production	[156]
SCID mice implanted with human tumor xenograft LAPC4	Prostate	FO	-	↓COX-2/PGE2 pathway	[158]
Athymic nude mice implanted with human tumor xenograft A549	Lung	DHA	-	↓EGFR onco-protein; ↓EGFR and ERK signaling	[117]
Fat-1 transgenic mice implanted with Lewis	Lung	-	-	↓AMK and PI3K/Akt singnaling: ↑autophagy and apoptosis	[133]
Athymic nude mice implanted with human tumor xenograft MIA-PaCa-2	Pancreatic	FO	-	↑ROS production; ↑autophagosome formation	[58]
Fat-1 transgenic mice implanted with PANC02	Pancreatic	-	-	↓Wnt/β-catenin signaling	[125]
Athymic nude mice implanted with human tumor xenograft COX-2 negative and positive BxPC-3	Pancreatic	FO	-	↓COX-2/PGE2 pathway, ↑PGE3	[159]
Athymic nude rats implanted with human tumor xenograft multi-drug resistant SK-*N-*BE(2)	Neuroblastoma	DHA	-	↑lipid peroxidation	[41]

Abbreviations: EPA, eicosapentaenoic acid; DHA, docosahexaenoic acid; FO, fish oil; PUFAs, polyunsatured fatty acids; HIF-1α, hypoxia-inducible factor 1-α; VEGF, vascular endothelial growth factor; MMPs, matrix metalloproteinases; COX, cyclooxygenase; Bcl, B-cell lymphoma; PI3K, phosphoinositide 3-kinase; NF-κB, nuclear factor-κB; PTEN, phosphatase and tensin homolog deleted on chromosome ten; ERK, extracellular signal-regulated kinase; EGFR, epidermal growth factor receptor; ROS, reactive oxygen species; PG, prostaglandin, GPx, glutathione peroxidase; AMK, adenosine monophosphate kinase; SCID, severe combined immunodeficiency; Ref., reference number.

**Figure 1:**
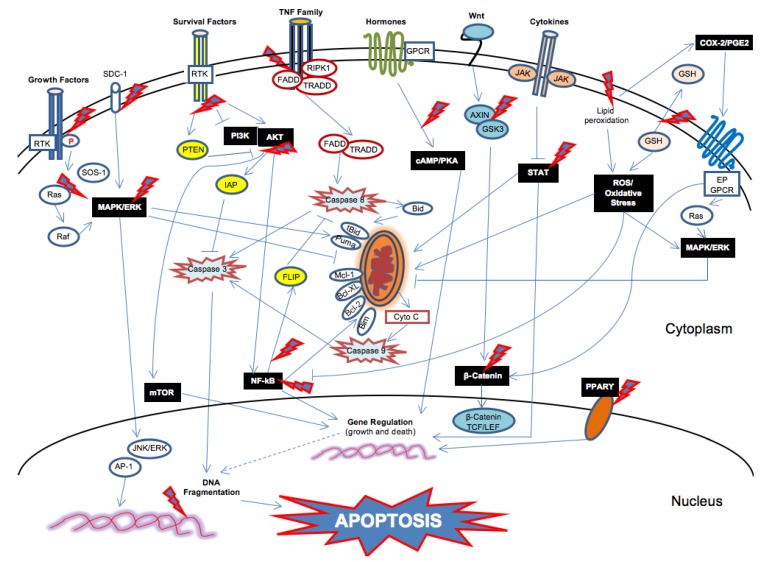
Multiple apoptotic molecular signals targeted by *n-*3 PUFAs in cancer cells. Abbreviations: RTK, protein tyrosine kinase; SOS-1, son of sevenless-1; Erk, extracellular-signal-regulated kinase; MAPK, mitoge*n-*activated protein kinase; JNK, Jun *N-*terminal kinase; AP-1, activator protei*n-*1; SDC-1, syndeca*n-*1; PTEN, phosphatase and tensin homolog deleted on chromosome ten; PI3K, phosphatidylinositol-3-kinase; mTOR, mammalian target of rapamycin; IAP, inhibitor of apoptosis; NF-κB, nuclear factor-κB; FLIP, FLICE-like inhibitory protein; RIPK-1, receptor-like protein kinase-1; FADD, Fas-associated death domain; TRADD, TNF receptor-associated death domain; Bcl-2, B-cell lymphoma protei*n-*2; Bim, Cyto C, cytochrome C; GPCR, G-protein coupled receptor; cAMP, cyclic adenosine monophosphate; PKA, protein kinase A; GSK, glycogen synthase kinase; TCF, T-cell factor; LEF, lymphoid enhancer-binding factor; JAK, Janus kinase; STAT, signal transducer and activator of transcription; ROS, reactive oxygen species; GSH, glutathione; COX-2, cyclooxygenase-2; PGE2, prostaglandin E2; PPAR-γ, peroxisome proliferator-activated receptor γ. arrows, activation; ┴, inhibition; dashed arrows, indirect action; red/blue flash, targeted by *n-*3 PUFAs.

## 4. Conclusions

The targeting of tumor cell apoptosis has important therapeutic potential. It is known that essentially all chemotherapeutic drugs and radiotherapy regimens that are in clinical use induce apoptosis of malignant cells when they work properly. However, the resistance to therapy, due to the modulation of the expression of multiple genes and gene products involved in cell death and survival, prompt oncologists to believe that, for a more effective apoptosis-based treatment, combinational therapies are needed to target multitude molecular signals involved in cancer cell death. Several studies have proposed the potential cability of *n-*3 PUFAs DHA and EPA to enhance the efficacy as well as the tolerability of conventional anticancer therapies. Taken together, the data presented in this review, showing the ability of *n-*3 PUFAs, DHA and EPA to induce cytotoxicity via apoptosis in different tumor cell types *in vitro* (Table 2) and *in vivo* (Table 3), indicate that these FAs potentially target multiple molecular signals involved in tumor cell death (Figure 1).

The use of multiple different pathways by *n-*3 PUFAs to trigger apoptosis in tumor cells may be partly related to the diverse activities possibly exerted in diverse cellular cancer models, but also to the different *n-*3 PUFAs used (EPA, DHA or FO), as well as to the different ways of administration, such as doses and kinetics. The context is complex and it might be even more complex if we consider that most of the molecular signals converge into the nucleus, altering gene expression. The pleiotropic nature of transcriptional changes induced by *n-*3 PUFAs have been recently illustrated by studies where global gene expression patterns were determined by microarray analysis *in vitro* and *in vivo* [44,166,167]. Several genes, potentially involved directly or indirectly in cancer cell apoptosis, appear to be regulated by *n-*3 PUFAs, underlining the complexity of the mechanisms involved in the induction of cancer cell apoptosis by these FAs. Therefore, further basic research is needed to show which pathways are crucial for the control of tumor cell apoptosis by *n-*3 PUFAs. Moreover, a clear need appears for further clinical studies, evaluating the potential role of DHA and EPA supplementation, mainly in combination with chemo- and radio-therapeutic anticancer regimens, in the improvement of patients’ clinical outcome and survival.

## References

[1] Ma X., Yu H. (2006). Global burden of cancer. J. Biol. Med..

[2] Basile K.J., Aplin A.E. (2012). Resistance to chemotherapy: Short-term drug tolerance and stem cell-like subpopulations. Adv. Pharmacol..

[3] Reya T., Morrison S.J., Clarke M.F., Weissman I.L. (2001). Stem cells, cancer, and cancer stem cells. Nature.

[4] Maugeri-Saccà M., Vigneri P., de Maria R. (2011). Cancer stem cells and chemosensitivity. Clin. Cancer Res..

[5] Hanahan D., Weinberg R.A. (2011). Hallmarks of cancer: The next generation. Cell.

[6] Pritchard J.R., Bruno P.M., Gilbert L.A., Capron K.L., Lauffenburger D.A., Hemann M.T. (2013). Defining principles of combination drug mechanisms of action. Proc. Natl. Acad. Sci. USA.

[7] Burlingame B., Nishida C., Uauy R., Weisell R. (2009). Fats and fatty acids in human nutrition: Introduction. Ann. Nutr. Metable.

[8] Riediger N.D., Othman R.A., Suh M., Moghadasian M.H. (2009). A systemic review of the roles of *n-*3 fatty acids in health and disease. J. Am. Diet. Assoc..

[9] Calder P.C. (2015). Marine ω-3 Fatty acids and inflammatory processes: Effects, mechanisms and clinical relevance. Biochim. Biophys. Acta.

[10] Gil A., Gil F. (2015). Fish, a Mediterranean source of *n-*3 PUFA: Benefits do not justify limiting consumption. Br. J. Nutr..

[11] Laviano A., Rianda S., Molfino A., Rossi Fanelli F. (2013). ω-3 Fatty acids in cancer. Curr. Opin. Clin. Nutr. Metab. Care.

[12] Bhagat U., Das U.N. (2015). Potential role of dietary lipids in the prophylaxis of some clinical conditions. Arch. Med. Sci..

[13] Murray M., Hraiki A., Bebawy M., Pazderka C., Rawling T. (2015). Anti-tumor activities of lipids and lipid analogues and their development as potential anticancer drugs. Pharmacol. Ther..

[14] Bang H.O., Dyerberg J., Nielsen A.B. (1971). Plasma lipid and lipoprotein pattern in Greenlandic West-coast Eskimos. Lancet.

[15] Gu Z., Shan K., Chen H., Chen Y.Q. (2015). *n-*3 Polyunsaturated fatty acids and their role in cancer chemoprevention. Curr. Pharmacol. Rep..

[16] Serini S., Fasano E., Piccioni E., Cittadini A.R., Calviello G. (2011). Dietary *n-*3 polyunsaturated fatty acids and the paradox of their health benefits and potential harmful effects. Chem. Res. Toxicol..

[17] Chapkin R.S., DeClercq V., Kim E., Fuentes N.R., Fan Y.Y. (2014). Mechanisms by which pleiotropic amphiphilic *n-*3 PUFA reduce colon cancer risk. Curr. Colorectal Cancer Rep..

[18] Kiyabu G.Y., Inoue M., Saito E., Abe S.K., Sawada N., Ishihara J., Iwasaki M., Yamaji T., Shimazu T. (2015). JPHC Study Group. Fish, *n*-3 polyunsaturated fatty acids and *n*-6 polyunsaturated fatty acids intake and breast cancer risk: The Japan Public Health Center-based prospective study. Int. J. Cancer..

[19] Brasky T.M., Darke A.K., Song X., Tangen C.M., Goodman P.J., Thompson I.M., Meyskens F.L., Goodman G.E., Minasian L.M. (2013). Plasma phospholipid fatty acids and prostate cancer risk in the SELECT trial. J. Natl. Cancer Inst..

[20] Calder P.C., Deckelbaum R.J. (2014). Dietary fatty acids in health and disease: Greater controversy, greater interest. Curr. Opin. Clin. Nutr. Metab. Care.

[21] Weylandt K.H., Serini S., Chen Y.Q., Su H.M., Lim K., Cittadini A., Calviello G. (2015). ω-3 Polyunsaturated fatty acids: The way forward in times of mixed evidence. Biomed. Res. Int..

[22] Berquin I.M., Edwards I.J., Chen Y.Q. (2008). Multi-targeted therapy of cancer by ω-3 Fatty acids. Cancer Lett..

[23] Serini S., Piccioni E., Merendino N., Calviello G. (2009). Dietary polyunsaturated fatty acids as inducers of apoptosis: Implications for cancer. Apoptosis.

[24] Gleissman H., Johnsen J.I., Kogner P. (2010). ω-3 Fatty acids in cancer, the protectors of good and the killers of evil?. Exp. Cell Res..

[25] Vaughan V.C., Hassing M.R., Lewandowski P.A. (2013). Marine polyunsaturated fatty acids and cancer therapy. Br. J. Cancer.

[26] Biondo P.D., Brindley D.N., Sawyer M.B., Field C.J. (2008). The potential for treatment with dietary long-chain polyunsaturated *n-*3 fatty acids during chemotherapy. J. Nutr. Biochem..

[27] Siddiqui R.A., Harvey K.A., Xu Z., Bammerlin E.M., Walker C., Altenburg J.D. (2011). Docosahexaenoic acid: A natural powerful adjuvant that improves efficacy for anticancer treatment with no adverse effects. Biofactors.

[28] Wang J., Luo T., Li S., Zhao J. (2012). The powerful applications of polyunsaturated fatty acids in improving the therapeutic efficacy of anticancer drugs. Expert Opin. Drug Deliv..

[29] Merendino N., Costantini L., Manzi L., Molinari R., D’Eliseo D., Velotti F. (2013). Dietary ω -3 polyunsaturated fatty acid DHA: A potential adjuvant in the treatment of cancer. Biomed. Res. Int..

[30] Hajjaji N., Bougnoux P. (2013). Selective sensitization of tumors to chemotherapy by marine-derived lipids: A review. Cancer Treat. Rev..

[31] de Aguiar Pastore Silva J., Emilia de Souza Fabre M., Waitzberg D.L. (2015). ω-3 Supplements for patients in chemotherapy and/or radiotherapy: A systematic review. Clin. Nutr..

[32] Das U.N., Madhavi N., Sravan Kumar G., Padma M., Sangeetha P. (1998). Can tumour cell drug resistance be reversed by essential fatty acids and their metabolites?. Prostaglandins Leukot. Essent. Fatty Acids.

[33] Slagsvold J.E., Pettersen C.H., Størvold G.L., Follestad T., Krokan H.E., Schønberg S.A. (2010). DHA alters expression of target proteins of cancer therapy in chemotherapy resistant SW620 colon cancer cells. Nutr. Cancer.

[34] Kuan C.Y., Walker T.H., Luo P.G., Chen C.F. (2011). Long-chain polyunsaturated fatty acids promote paclitaxel cytotoxicity via inhibition of the MDR1 gene in the human colon cancer Caco-2 cell line. J. Am. Coll. Nutr..

[35] Gelsomino G., Corsetto P.A., Campia I., Montorfano G., Kopecka J., Castella B., Gazzano E., Ghigo D., Rizzo A.M., Riganti C. (2013). ω 3 Fatty acids chemosensitize multidrug resistant colon cancer cells by dow*n-*regulating cholesterol synthesis and altering detergent resistant membranes composition. Mol. Cancer.

[36] Das U.N., Begin M.E., Ells G., Huang Y.S., Horrobin D.F. (1987). Polyunsaturated fatty acids augment free radical generation in tumor cells *in vitro*. Biochem. Biophys. Res. Commun..

[37] Tsai W.S., Nagawa H., Kaizaki S., Tsuruo T., Muto T. (1998). Inhibitory effects of *n-*-3 polyunsaturated fatty acids on sigmoid colon cancer transformants. J. Gastroenterol..

[38] Siddiqui R.A., Harvey K., Stillwell W. (2008). Anticancer properties of oxidation products of docosahexaenoic acid. Chem. Phys. Lipids.

[39] Giros A., Grzybowski M., Sohn V.R., Pons E., Fernandez-Morales J., Xicola R.M., Sethi P., Grzybowski J., Goel A. (2009). Regulation of colorectal cancer cell apoptosis by the *n-*3 polyunsaturated fatty acids Docosahexaenoic and Eicosapentaenoic. Cancer Prev. Res..

[40] Toit-Kohn J.L., Louw L., Engelbrecht A.M. (2009). Docosahexaenoic acid induces apoptosis in colorectal carcinoma cells by modulating the PI3 kinase and p38 MAPK pathways. J. Nutr. Biochem..

[41] Gleissman H., Segerström L., Hamberg M., Ponthan F., Lindskog M., Johnsen J.I., Kogner P. (2011). ω-3 Fatty acid supplementation delays the progression of neuroblastoma *in vivo*. Int. J. Cancer.

[42] Nikolakopoulou Z., Nteliopoulos G., Michael-Titus A.T., Parkinson E.K. (2013). ω-3 Polyunsaturated fatty acids selectively inhibit growth in neoplastic oral keratinocytes by differentially activating ERK1/2. Carcinogenesis.

[43] Ravacci G.R., Brentani M.M., Tortelli T.Jr., Torrinhas R.S., Saldanha T., Torres E.A., Waitzberg D.L. (2013). Lipid raft disruption by docosahexaenoic acid induces apoptosis in transformed human mammary luminal epithelial cells harboring HER-2 overexpression. J. Nutr. Biochem..

[44] Abdi J., Garssen J., Faber J., Redegeld F.A. (2014). ω-3 Fatty acids, EPA and DHA induce apoptosis and enhance drug sensitivity in multiple myeloma cells but not in normal peripheral mononuclear cells. J. Nutr. Biochem..

[45] Berstad P., Thiis-Evensen E., Vatn M.H., Almendingen K. (2012). Fatty acids in habitual diet, plasma phospholipids, and tumour and normal colonic biopsies in young colorectal cancer patients. J. Oncol..

[46] Thomas G.C. (2009). Apoptosis and cancer: The genesis of a research field. Nature Rev. Cancer.

[47] Logue S.E., Gorman A.M., Cleary P., Keogh N., Samali A. (2013). Current concepts in ER stress-induced apoptosis. J. Carcinogene Mutagene.

[48] Mengeaud V., Nano J.L., Fournel S., Rampal P. (1992). Effects of eicosapentaenoic acid, γ-linolenic acid and prostaglandin E1 on three human colon carcinoma cell lines. Prostaglandins Leukot. Essent. Fatty Acids.

[49] Clarke R.G., Lund E.K., Latham P., Pinder A.C., Johnson I.T. (1999). Effect of eicosapentaenoic acid on the proliferation and incidence of apoptosis in the colorectal cell line HT29. Lipids.

[50] Chen Z.Y., Istfan N.W. (2000). Docosahexaenoic acid is a potent inducer of apoptosis in HT-29 colon cancer cells. Prostaglandins Leukot. Essent. Fatty Acids.

[51] Kubota H., Matsumoto H., Higashida M., Murakami H., Nakashima H., Oka Y., Okumura H., Yamamura M., Nakamura M., Hirai T. (2013). Eicosapentaenoic acid modifies cytokine activity and inhibits cell proliferation in an oesophageal cancer cell line. Anticancer Res..

[52] Lee S.E., Lim J.W., Kim H. (2009). Activator protei*n-*1 mediates docosahexaenoic acid-induced apoptosis of human gastric cancer cells. Ann. N. Y. Acad. Sci..

[53] Lim K., Han C., Dai Y., Shenm M., Wu T. (2009). ω-3 Polyunsaturated fatty acids inhibit hepatocellular carcinoma cell growth through blocking β-catenin and cyclooxygenase-2. Mol. Cancer Ther..

[54] Sun S.N., Jia W.D., Chen H., Ma J.L., Ge Y.S., Yu J.H., Li J.S. (2013). Docosahexaenoic acid (DHA) induces apoptosis in human hepatocellular carcinoma cells. Int. J. of Clin. Exp. Pathol..

[55] Zhang Y., Han L., Qi W., Cheng D., Ma X., Hou L., Cao X., Wang C. (2015). Eicosapentaenoic acid (EPA) induced apoptosis in HepG2 cells through ROS-Ca^2+^-JNK mitochondrial pathways. Biochem. Biophys. Res. Commun..

[56] Hawkins R.A., Sangster K., Arends M.J. (1998). Apoptotic death of pancreatic cancer cells induced by polyunsaturated fatty acids varies with double bond number and involves an oxidative mechanism. J. Pathol..

[57] Merendino N., Loppi B., D’Aquino M., Molinari R., Pessina G., Romano C., Velotti F. (2005). Docosahexaenoic acid induces apoptosis in the human PaCa-44 pancreatic cancer cell line by active reduced glutathione extrusion and lipid peroxidation. Nutr. Cancer.

[58] Fukui M., Kang K.S., Okada K., Zhu B.T. (2013). EPA, an ω-3 Fatty acid, induces apoptosis in human pancreatic cancer cells: Role of ROS accumulation, caspase-8 activation, and autophagy induction. J. Cell. Biochem..

[59] Lim K., Han C., Xu L., Isse K., Demetris A.J., Wu T. (2008). Cyclooxygenase-2-derived prostaglandin E2 activates β-catenin in human cholangiocarcinoma cells: Evidence for inhibition of these signaling pathways by ω 3 polyunsaturated fatty acids. Cancer Res..

[60] Rose D.P., Connolly J.M. (1990). Effects of fatty acids and inhibitors of eicosanoid synthesis on the growth of a human breast cancer cell line in culture. Cancer Res..

[61] Chamras H., Ardashian A., Heber D., Glaspy J.A. (2002). Fatty acid modulation of MCF-7 human breast cancer cell proliferation, apoptosis and differentiation. J. Nutr. Biochem..

[62] Sharma A., Belna J., Logan J., Espat J., Hurteau J.A. (2005). The effects of ω-3 fatty acids on growth regulation of epithelial ovarian cancer cell lines. Gynecol. Oncol..

[63] Narayanan N.K., Narayanan B.A., Reddy B.S. (2005). A combination of docosahexaenoic acid and celecoxib prevents prostate cancer cell growth *in vitro* and is associated with modulation of nuclear factor-κB, and steroid hormone receptors. Int. J. Oncol..

[64] Hu Y., Sun H., Owens R.T., Gu Z., Wu J., Chen Y.Q., O’Flaherty J.T., Edwards I.J. (2010). Syndeca*n-*1-dependent suppression of PDK1/Akt/bad signaling by docosahexaenoic acid induces apoptosis in prostate cancer. Neoplasia.

[65] Molinari R., D’Eliseo D., Manzi L., Zolla L., Velotti F., Merendino N. (2011). The *n*3-polyunsaturated fatty acid docosahexaenoic acid induces immunogenic cell death in human cancer cell lines via pre-apoptotic calreticulin exposure. Cancer Immunol. Immunother..

[66] Lindskog M., Gleissman H., Ponthan F., Castro J., Kogner P., Johnsen J.I. (2006). Neuroblastoma cell death in response to docosahexaenoic acid: Sensitization to chemotherapy and arsenic induced oxidative stress. Int. J. Cancer.

[67] Faragó N., Fehér L.Z., Kitajka K., Das U.N., Puskás L.G. (2011). MicroRNA profile of polyunsaturated fatty acid treated glioma cells reveal apoptosis-specific expression changes. Lipids Health Dis..

[68] Serini S., Trombino S., Oliva F., Piccioni E., Monego G., Resci F., Boninsegna A., Picci N., Ranelletti F.O., Calviello G. (2008). Docosahexaenoic acid induces apoptosis in lung cancer cells by increasing MKP-1 and dow*n-*regulating p-ERK1/2 and p-p38 expression. Apoptosis.

[69] Yao Q.H., Zhang X.C., Fu T., Gu J.Z., Wang L., Wang Y., Lai Y.B., Wang Y.Q., Guo Y. (2014). ω-3 polyunsaturated fatty acids inhibit the proliferation of the lung adenocarcinoma cell line A549 *in vitro*. Mol. Med. Rep..

[70] Albino A.P., Juan G., Traganos F., Reinhart L., Connolly J., Rose D.P., Darzynkiewicz Z. (2000). Cell cycle arrest and apoptosis of melanoma cells by docosahexaenoic acid: Association with decreased pRb phosphorylation. Cancer Res..

[71] Denkins Y., Kempf D., Ferniz M., Nileshwar S., Marchetti D. (2005). Role of ω-3 polyunsaturated fatty acids on cyclooxygenase-2 metabolism in brai*n-*metastatic melanoma. J. Lipid Res..

[72] Finstad H.S., Myhrstad M.C., Heimli H., Lømo J., Blomhoff H.K., Kolset S.O., Drevon C.A. (1998). Multiplication and death-type of leukemia cell lines exposed to very long-chain polyunsaturated fatty acids. Leukemia.

[73] Finstad H.S., Drevon C.A., Kulseth M.A., Synstad A.V., Knudsen E., Kolset S.O. (1998). Cell proliferation, apoptosis and accumulation of lipid droplets in U937-1 cells incubated with eicosapentaenoic acid. Biochem. J..

[74] Chiu L.C.M., Wan J.M.F. (1999). Induction of apoptosis in HL-60 cells by eicosapentaenoic acid (EPA) is associated with downregulation of BCL-2 expression. Cancer Letters.

[75] Chiu L.C., Wong E.Y., Ooi V.E. (2004). Docosahexaenoic acid modulates different genes in cell cycle and apoptosis to control growth of human leukemia HL-60 cells. Int J Oncol..

[76] Siddiqui R.A., Jenski L.J., Neff K., Harvey K., Kovacs R.J., Stillwell W. (2001). Docosahexaenoic acid induces apoptosis in Jurkat cells by a protein phosphatase-mediated process. Biochim. Biophys. Acta.

[77] Zand H., Rhimipour A., Bakhshayesh M., Shafiee M., Nour Mohammadi I., Salimi S. (2007). Involvement of PPAR-γ and p53 in DHA-induced apoptosis in Reh cells. Mol. Cell. Biochem..

[78] Yamagami T., Porada C.D., Pardini R.S., Zanjani E.D., Almeida-Porada G. (2009). Docosahexaenoic acid induces dose dependent cell death in an early undifferentiated subtype of acute myeloid leukemia cell line. Cancer Biol. Ther..

[79] Sravan Kumar G., Das U.N. (1997). Cytotoxic action of α-linolenic and eicosapentaenoic acids on myeloma cells *in vitro*. Prostaglandins Leukot. Essent. Fatty Acids.

[80] Ricci-Vitiani L., Lombardi D.G., Pilozzi E., Biffoni M., Todaro M., Peschle C., de Maria R. (2007). Identification and expansion of human colo*n-*cancer-initiating cells. Nature.

[81] Das U.N. (2011). Essential fatty acids and their metabolites as modulators of stem cell biology with reference to inflammation, cancer, and metastasis. Cancer Metastasis Rev..

[82] Yang T., Fang S., Zhang H.X., Xu L.X., Zhang Z.Q., Yuan K.T., Xue C.L., Yu H.L., Zhang S., Li Y.F. (2013). *n-*3 PUFA shave antiproliferative and apoptotic effects on human colorectal cancer stemlike cells *in vitro*. J. Nutr. Biochem..

[83] Vasudevan A., Yu Y., Banerjee S., Woods J., Farhana L., Rajendra S.G., Patel A., Dyson G., Levi E., Maddipati K.R. (2014). ω-3 Fatty acid is a potential preventive agent for recurrent colon cancer. Cancer Prev. Res..

[84] De Carlo F., Witte T.R., Hardman W.E., Claudio P.P. (2013). ω-3 Eicosapentaenoic acid decreases CD133 colon cancer stem-like cell marker expression while increasing sensitivity to chemotherapy. PLoS ONE.

[85] Xiong A., Yu W., Liu Y., Sanders B.G., Kline K. (2015). Elimination of ALDH+ breast tumor initiating cells by docosahexanoic acid and/or γ tocotrienol through SHP-1 inhibition of Stat3 signaling. Mol. Carcinog..

[86] Rose D.P., Connolly J.M., Rayburn J., Coleman M. (1995). Influence of diets containing eicosapentaenoic or docosahexaenoic acid on growth and metastasis of breast cancer cells in nude mice. J. Natl. Cancer Inst..

[87] Yam D., Peled A., Huszar M., Shinitzky M. (1997). Dietary fish oil suppresses tumor growth and metastasis of Lewis lung carcinoma in mice. J. Nutr. Biochem..

[88] Boudreau M.D., Sohn K.H., Rhee S.H., Lee S.W., Hunt J.D., Hwang D.H. (2001). Suppression of tumor cell growth both in nude mice and in culture by *n-*3 polyunsaturated fatty acids: Mediation through cyclooxygenase-independent pathways. Cancer Res..

[89] Kato T., Hancock R.L., Mohammadpour H., McGregor B., Manalo P., Khaiboullina S., Hall M.R., Pardini L., Pardini R.S. (2002). Influence of ω-3 fatty acids on the growth of human colon carcinoma in nude mice. Cancer Lett..

[90] Camargo C.Q., Mocellin M.C., Pastore Silva J.A., de Souza Fabre M.E., Nunes E.A., de Moraes Trinidade E.B. Fish oil supplementation during chemotherapy increases posterior time to tumor progression in colorectal cancer. Nutr. Cancer.

[91] Bougnoux P., Hajjaji N., Ferrasson M.N., Giraudeau B., Couet C., le Floch O. (2009). Improving outcome of chemotherapy of metastatic breast cancer by docosahexaenoic acid: A phase II trial. Br. J. Cancer.

[92] Cockbain J., Toogood G.J., Hull M.A. (2012). ω-3 Polyunsaturated fatty acids for the treatment and prevention of colorectal cancer. Gut.

[93] Murphy R.A., Mourtzakis M., Chu Q.S., Baracos V.E., Reiman T., Mazurak V.C. (2011). Supplementation with fish oil increases first-line chemotherapy efficacy in patients with advanced no*n-*small cell lung cancer. Cancer.

[94] Patterson R.E., Flatt S.W., Newman V.A., Natarajan L., Rock C.L., Thomson C.A., Caan B.J., Parker B.A., Pierce J.P. (2011). Marine fatty acid intake is associated with breast cancer prognosis. J. Nutr..

[95] Cockbain A.J., Volpato M., Race A.D., Munarini A., Fazio C., Belluzzi A., Loadman P.M., Toogood G.J., Hull M.A. (2014). Anticolorectal cancer activity of the ω-3 polyunsaturated fatty acid eicosapentaenoic acid. Gut.

[96] Sánchez-Lara K., Turcott J.G., Juárez-Hernández E., Nuñez-Valencia C., Villanueva G., Guevara P., de la Torre-Vallejo M., Mohar A., Arrieta O. (2014). Effects of an oral nutritional supplement containing eicosapentaenoic acid on nutritional and clinical outcomes in patients with advanced no*n-*small cell lung cancer: Randomised trial. Clin. Nutr..

[97] Arshad A., Chung W.Y., Isherwood J., Mann C.D., Al-Leswas D., Steward W.P., Metcalfe M.S., Dennison A.R. (2014). Cellular and plasma uptake of parenteral ω-3 rich lipid emulsion fatty acids in patients with advanced pancreatic cancer. Clin. Nutr..

[98] Ma Y.J., Yu J., Xiao J., Cao B.W. (2015). The consumption of ω-3 polyunsaturated fatty acids improves clinical outcomes and prognosis in pancreatic cancer patients: A systematic evaluation. Nutr. Cancer.

[99] Nabavi S.F., Bilottom S., Russom G.L., Orhan I.E., Habtemariam S., Daglia M., Devi K.P., Loizzo M.R., Tundis R., Nabavi S.M. (2015). ω-3 polyunsaturated fatty acids and cancer: Lessons learned from clinical trials. Cancer Metastasis Rev..

[100] Khankari N.K., Bradshaw P.T., Steck S.E., He K., Olshan A.F., Shen J., Ahn J., Chen Y., Ahsan H., Terry M.B. (2015). Dietary intake of fish, polyunsaturated fatty acids, and survival after breast cancer: A populatio*n-*based follow-up study on Long Island, New York. Cancer.

[101] Mocellin M.C., Camargo C.Q., Nunes E.A., Fiates G.M., Trindade E.B. (2015). A systematic review and meta-analysis of the *n-*3 polyunsaturated fatty acids effects on inflammatory markers in colorectal cancer. Clin. Nutr..

[102] Heimli H., Giske C., Naderi S., Drevon C.A., Hollung K. (2002). Eicosapentaenoic acid promotes apoptosis in Ramos cells via activation of caspase-3 and -9. Lipids.

[103] Llor X., Pons E., Roca A., Alvarez M., Mañé J., Fernández-Bañares F., Gassull M.A. (2003). The effects of fish oil, olive oil, oleic acid and linoleic acid on colorectal neoplastic processes. Clin. Nutr..

[104] Danbara N., Yuri T., Tsujita-Kyutoku M., Sato M., Senzaki H., Takada H., Hada T., Miyazawa T., Okazaki K., Tsubura A. (2004). Conjugated docosahexaenoic acid is a potent inducer of cell cycle arrest and apoptosis and inhibits growth of colo 201 human colon cancer cells. Nutr. Cancer.

[105] Calviello G., Di Nicuolo F., Serini S., Piccioni E., Boninsegna A., Maggiano N., Ranelletti F.O., Palozza P. (2005). Docosahexaenoic acid enhances the susceptibility of human colorectal cancer cells to 5-fluorouracil. Cancer Chemother. Pharmacol..

[106] Jakobsen C.H., Størvold G.L., Bremseth H., Follestad T., Sand K., Mack M., Olsen K.S., Lundemo A.G., Iversen J.G., Krokan H.E. (2008). DHA induces ER stress and growth arrest in human colon cancer cells: Associations with cholesterol and calcium homeostasis. J. Lipid. Res..

[107] Arita K., Kobuchi H., Utsumi T., Takehara Y., Akiyama J., Horton A.A., Utsumi K. (2001). Mechanism of apoptosis in HL-60 cells induced by *n-*3 and *n-*6 polyunsaturated fatty acids. Biochem. Pharmacol..

[108] Narayanan B.A., Narayanan N.K., Reddy B.S. (2001). Docosahexaenoic acid regulated genes and transcription factors inducing apoptosis in human colon cancer cells. Int. J. Oncol..

[109] Kolch W., Halasz M., Granovskaya M., Kholodenko B.N. (2015). The dynamic control of signal transduction networks in cancer cells. Nat. Rev. Cancer.

[110] Huang C.Y., Yu L.C. (2015). Pathophysiological mechanisms of death resistance in colorectal carcinoma. World J. Gastroenterol..

[111] Glatz J.F., Luiken J.J., van Nieuwenhoven F.A., van der Vusse G.J. (1997). Molecular mechanism of cellular uptake and intracellular translocation of fatty acids. Prostaglandins Leukot. Essent. Fatty Acids.

[112] Wassall S.R., Stillwell W. (2009). Polyunsaturated fatty acid-cholesterol interactions: Domain formation in membranes. Biochim. Biophys. Acta.

[113] Zhang C., Yu H., Ni X., Shen S., Das U.N. (2015). Growth inhibitory effect of polyunsaturated fatty acids (PUFAs) on colon cancer cells via their growth inhibitory metabolites and fatty acid composition changes. PLoS ONE.

[114] Ibarguren M., López D.J., Escribá P.V. (2014). The effect of natural and synthetic fatty acids on membrane structure, microdomain organization, cellular functions and human health. Biochim. Biophys. Acta.

[115] Corsetto P.A., Cremona A., Montorfano G., Jovenitti I.E., Orsini F., Arosio P., Rizzo A.M. (2012). Chemical-physical changes in cell membrane microdomains of breast cancer cells after ω-3 PUFA incorporation. Cell Biochem. Biophys..

[116] Schley P.D., Brindley D.N., Field C.J. (2007). (*n-*3) PUFA alter raft lipid composition and decrease epidermal growth factor receptor levels in lipid rafts of human breast cancer cells. J. Nutr..

[117] Rogers K.R., Kikawa K.D., Mouradian M., Hernandez K., McKinnon K.M., Ahwah S.M., Pardini R.S. (2010). Docosahexaenoic acid alters epidermal growth factor receptor-related signaling by disrupting its lipid raft association. Carcinogenesis.

[118] Corsetto P.A., Montorfano G., Zava S., Jovenitti I.E., Cremona A., Berra B., Rizzo A.M. (2011). Effects of *n-*3 PUFAs on breast cancer cells through their incorporation in plasma membrane. Lipids Health Dis..

[119] Lee E.J., Yun U.J., Koo K.H., Sung J.Y., Shim J., Ye S.K., Hong K.M., Kim Y.N. (2014). Dow*n-*regulation of lipid raft-associated onco-proteins via cholesterol-dependent lipid raft internalization in docosahexaenoic acid-induced apoptosis. Biochim. Biophys. Acta.

[120] Mason J.K., Klaire S., Kharotia S., Wiggins A.K., Thompson L.U. (2015). α-linolenic acid and docosahexaenoic acid, alone and combined with trastuzumab, reduce HER2-overexpressing breast cancer cell growth but differentially regulate HER2 signaling pathways. Lipids Health Dis..

[121] Cao W., Ma Z., Rasenick M.M., Yeh S., Yu J. (2012). *n-*3 poly-unsaturated fatty acids shift estrogen signaling to inhibit human breast cancer cell growth. PLoS ONE.

[122] Ewaschuk J.B., Newell M., Field C.J. (2012). Docosahexanoic acid improves chemotherapy efficacy by inducing CD95 translocation to lipid rafts in ER^−^ breast cancer cells. Lipids.

[123] Gu Z., Wu J., Wang S., Suburu J., Chen H., Thomas M.J., Shi L., Edwards I.J., Berquin I.M., Chen Y.Q. (2013). Polyunsaturated fatty acids affect the localization and signaling of PIP3/AKT in prostate cancer cells. Carcinogenesis.

[124] Calviello G., Resci F., Serini S., Piccioni E., Toesca A., Boninsegna A., Monego G., Ranelletti F.O., Palozza P. (2007). Docosahexaenoic acid induces proteasome-dependent degradation of β-catenin, dow*n-*regulation of survivin and apoptosis in human colorectal cancer cells not expressing COX-2. Carcinogenesis.

[125] Song K.S., Jing K., Kim J.S., Yun E.J., Shin S., Seo K.S., Park J.H., Heo J.Y., Kang J.X., Suh K.S. (2011). ω-3-Polyunsaturated fatty acids suppress pancreatic cancer cell growth *in vitro* and *in vivo* via downregulation of Wnt/β-catenin signaling. Pancreatology.

[126] Xue M., Wang Q., Zhao J., Dong L., Ge Y., Hou L., Liu Y., Zheng Z. (2014). Docosahexaenoic acid inhibited the Wnt/β-catenin pathway and suppressed breast cancer cells *in vitro* and *in vivo*. J. Nutr. Biochem..

[127] Sun H., Hu Y., Gu Z., Owens R.T., Chen Y.Q., Edwards I.J. (2011). ω-3 Fatty acids induce apoptosis in human breast cancer cells and mouse mammary tissue through syndecan*-*1 inhibition of the MEK-Erk pathway. Carcinogenesis.

[128] Schley P.D., Jijon H.B., Robinson L.E., Field C.J. (2005). Mechanisms of ω-3 fatty acid-induced growth inhibition in MDAMB-231 human breast cancer cells. Breast Cancer Res. Treat..

[129] Ghosh-Choudhury T., Mandal C.C., Woodruff K., St Clair P., Fernandes G., Choudhury G.G., Ghosh-Choudhury N. (2009). Fish oil targets PTEN to regulate NFκB for downregulation of anti-apoptotic genes in breast tumor growth. Breast Cancer Res. Treat..

[130] Engelbrecht A.M., Toit-Kohn J.L., Ellis B., Thomas M., Nell T., Smith R. (2008). Differential induction of apoptosis and inhibition of the PI3-kinase pathway by saturated, monounsaturated and polyunsaturated fatty acids in a colon cancer cell model. Apoptosis.

[131] Jing K., Song K.S., Shin S., Kim N., Jeong S., Oh H.R., Park J.H., Seo K.S., Heo J.Y., Han J. (2011). Docosahexaenoic acid induces autophagy through p53/AMPK/mTOR signaling and promotes apoptosis in human cancer cells harboring wild-type p53. Autophagy.

[132] Shin S., Jing K., Jeong S., Kim N., Song K.S., Heo J.Y., Park J.H., Seo K.S., Han J., Park J.I. (2013). The ω-3 polyunsaturated fatty acid DHA induces simultaneous apoptosis and autophagy via mitochondrial ROS-mediated Akt-mTOR signaling in prostate cancer cells expressing mutant p53. Biomed. Res. Int..

[133] Kim N., Jeong S., Jing K., Shin S., Kim S., Heo J.Y., Kweon G.R., Park S.K., Wu T., Park J.I. (2015). Docosahexaenoic acid induces cell death in human no*n-*small cell Lung cancer cells by repressing mTOR via AMPK activation and PI3K/Akt inhibition. Biomed. Res. Int..

[134] Rescigno T., Capasso A., Tecce M.F. (2015). Effect of Docosahexaenoic acid on cell cycle pathways in Breast cell lines with different transformation degree. J. Cell. Physiol..

[135] Shaikh I.A., Brown I., Schofield A.C., Wahle K.W., Heys S.D. (2008). Docosahexaenoic acid enhances the efficacy of docetaxel in prostate cancer cells by modulation of apoptosis: The role of genes associated with the NF-κB pathway. Prostate.

[136] Cavazos D.A., Price R.S., Apte S.S., de Graffenried L.A. (2011). Docosahexaenoic acid selectively induces human prostate cancer cell sensitivity to oxidative stress through modulation of NF-κB. Prostate.

[137] Jeong S., Jing K., Kim N., Shin S., Kim S., Song K.S., Heo J.Y., Park J.H., Seo K.S., Han J. (2014). Docosahexaenoic acid-induced apoptosis is mediated by activation of mitoge*n-*activated protein kinases in human cancer cells. BMC Cancer.

[138] Gleissman H., Yang R., Martinod K., Lindskog M., Serhan C.N., Johnsen J.I., Kogner P. (2010). Docosahexaenoic acid metabolome in neural tumors: Identification of cytotoxic intermediates. FASEB J..

[139] Serhan C.N., Arita M., Hong S., Gotlinger K. (2004). Resolvins, docosatrienes, and neuroprotectins, novel ω-3-derived mediators, and their endogenous aspiri*n-*triggered epimers. Lipids.

[140] Hong S., Lu Y., Yang R., Gotlinger K.H., Petasis N.A., Serhan C.N. (2007). Resolvin D1, protectin D1, and related docosahexaenoic acid-derived products: Analysis via electrospray/low energy tandem mass spectrometry based on spectra and fragmentation mechanisms. J. Am. Soc. Mass Spectrom..

[141] Ding W.Q., Vaught J.L., Yamauchi H., Lind S.E. (2004). Differential sensitivity of cancer cells to docosahexaenoic acid-induced cytotoxicity: The potential importance of dow*n-*regulation of superoxide dismutase 1 expression. Mol. Cancer Ther..

[142] Ding W.Q., Lind S.E. (2007). Phospholipid hydroperoxide glutathione peroxidase plays a role in protecting cancer cells from docosahexaenoic acid-induced cytotoxicity. Mol. Cancer Ther..

[143] Vibet S., Goupille C., Bougnoux P., Steghens J.P., Goré J., Mahéo K. (2008). Sensitization by docosahexaenoic acid (DHA) of breast cancer cells to anthracyclines through loss of glutathione peroxidase (GPx1) response. Free Radic. Biol. Med..

[144] Sturlan S., Baumgartner M., Roth E., Bachleitner-Hofmann T. (2003). Docosahexaenoic acid enhances arsenic trioxidemediated apoptosis in arsenic trioxide-resistant HL-60 cells. Blood.

[145] Granci V., Cai F., Lecumberri E., Clerc A., Dupertuis Y.M., Pichar C. (2013). Colon cancer cell chemosensitisation by fish oil emulsion involves apoptotic mitochondria pathway. Br. J. Nutr..

[146] Hossain Z., Hosokawa M., Takahashi K. (2009). Growth inhibition and induction of apoptosis of colon cancer cell lines by applying marine phospholipid. Nutrition and Cancer.

[147] Dai J., Shen J., Pan W., Shen S., Das U.N. (2013). Effects of polyunsaturated fatty acids on the growth of gastric cancer cells *in vitro*. Lipids Health Dis..

[148] Jing K., Shin S., Jeong S., Kim S., Song K.S., Park J.H., Heo J.Y., Seo K.S., Park S.K., Kweon G.R. (2014). Docosahexaenoic acid induces the degradation of HPV E6/E7 oncoproteins by activating the ubiquiti*n-*proteasome system. Cell Death Dis..

[149] Kang K.S., Wang P., Yamabe N., Fukui M., Jay T., Zhu B.T. (2010). Docosahexaenoic acid induces apoptosis in MCF-7 cells *in vitro* and *in vivo* via reactive oxygen species formation and caspase 8 activation. PLoS ONE.

[150] Zajdel A., Wilczok A., Tarkowski M. (2015). Toxic effects of *n-*3 polyunsaturated fatty acids in human lung A549 cells. Toxicol. Vitro.

[151] Serhan C.N., Hong S., Gronert K., Colgan S.P., Devchand P.R., Mirick G., Moussignac R.L. (2002). Resolvins: A family of bioactive products of ω-3 fatty acid transformation circuits initiated by aspirin treatment that counter proinflammation signals. J. Exp. Med..

[152] Wang Q., Hu M., Xu H., Yang X. (2016). Anti-inflammatory and Pro-resolving effects of *n-*3 PUFA in Cancers: Structures and Mechanisms. Curr. Top Med. Chem..

[153] Hawcroft G., Loadman P.M., Belluzzi A., Hull M.A. (2010). Effect of eicosapentaenoic acid on E-type prostaglandin synthesis and EP4 receptor signaling in human colorectal cancer cells. Neoplasia.

[154] Poorani R., Bhatt A.N., Dwarakanath B.S., Das U.N. (2015). COX-2, aspirin and metabolism of arachidonic, eicosapentaenoic and docosahexaenoic acids and their physiological and clinical significance. Eur. J. Pharmacol..

[155] Karmali R.A., Reichel P., Cohen L.A., Terano T., Hirai A., Tamura Y., Yoshida S. (1987). The effects of dietary ω−3 fatty acids on the DU-145 transplantable human prostatic tumor. Anticancer Res..

[156] Rose D.P., Cohen L.A. (1988). Effects of dietary menhaden oil and retinyl acetate on the growth of DU145 human prostatic adenocarcinoma cells transplanted into athymic nude mice. Carcinogenesis.

[157] Rose D.P., Connolly J.M. (1997). Dietary fat and breast cancer metastasis by human tumor xenografts. Breast Cancer Res. Treat..

[158] Kobayashi N., Barnard R.J., Henning S.M., Elashoff D., Reddy S.T., Cohen P., Leung P., Hong-Gonzalez J., Freedland S.J., Said J. (2006). Effect of altering dietary ω-6/ω-3 fatty acid ratios on prostate cancer membrane composition, cyclooxygenase-2, and prostaglandin E_2_. Clin. Cancer Res..

[159] Funahashi H., Satake M., Hasan S., Sawai H., Newman R.A., Reber H.A., Hines O.J., Eibl G. (2008). Opposing effects of *n-*6 and *n-*3 polyunsaturated fatty acids on pancreatic cancer growth. Pancreas.

[160] Narayanan B.A., Narayanan N.K., Desai D., Pittman B., Reddy B.S. (2004). Effects of a combination of docosahexaenoic acid and 1,4-phenylene bis(methylene) selenocyanate on cyclooxygenase 2, inducible nitric oxide synthase and β-catenin pathways in colon cancer cells. Carcinogenesis.

[161] Calviello G., Serini S., Piccioni E. (2007). *n-*3 polyunsaturated fatty acids and the prevention of colorectal cancer: Molecular mechanisms involved. Curr. Med. Chem..

[162] Sun H., Berquin I.M., Owens R.T., O’Flaherty J.T., Edwards I.J. (2008). Peroxisome proliferator-activated receptor γ-mediated up-regulation of syndeca*n-*1 by *n-*3 fatty acids promotes apoptosis of human breast cancer cells. Cancer Res..

[163] Edwards I.J., Sun H., Hu Y., Berquin I.M., O’Flaherty J.T., Cline J.M., Rudel L.L., Chen Y.Q. (2008). *In vivo* and *in vitro* regulation of syndecan 1 in prostate cells by *n-*3 polyunsaturated fatty acids. J. Biol. Chem..

[164] O’Flaherty J.T., Hu Y., Wooten R.E., Horita D.A., Samuel M.P., Thomas M.J., Sun H., Edwards I.J. (2012). 15-lipoxygenase metabolites of docosahexaenoic acid inhibit prostate cancer cell proliferation and survival. PLoS ONE.

[165] Hu Y., Sun H., O’Flaherty J.T., Edwards I.J. (2013). 15-Lipoxygenase-1-mediated metabolism of docosahexaenoic acid is required for syndeca*n-*1 signaling and apoptosis in prostate cancer cells. Carcinogenesis.

[166] Zou S., Meng X., Meng Y., Liu J., Liu B., Zhang S., Ding W., Wu J., Zhou J. (2015). Microarray analysis of anti-cancer effects of docosahexaenoic acid on human colon cancer model in nude mice. Int. J. Clin. Exp. Med..

[167] Sheng H., Li P., Chen X., Liu B., Zhu Z., Cao W. (2014). ω-3 PUFAs induce apoptosis of gastric cancer cells via ADORA1. Front. Biosci..

